# Dual-band patch antenna for 5G NR (n48, n46, n77, n78), Wi-Fi, and indoor wireless applications

**DOI:** 10.1038/s41598-026-41860-1

**Published:** 2026-03-18

**Authors:** Prem Pal Singh, Vishal Sorathiya, Fahad Ahmed Al-zahrani

**Affiliations:** 1 Faculty of Engineering and Technology, Parul Institute of Engineering and Technology, Parul University, Vadodara, Gujarat India; 2 Computer Engineering Department, Umm Al-Qura University, 24381 Mecca, Saudi Arabia

**Keywords:** Two-port, MIMO, 5G-NR applications, Wi-Fi, Energy science and technology, Engineering

## Abstract

This work presents a 2-port dual-band patch antenna operating in the sub-6 GHz range, suitable for various applications, including 5G, Wi-Fi, and other indoor wireless uses. It comprises two antenna elements arranged in parallel. The antenna functions effectively across two principal frequency bands: 3.50–3.68 GHz, with simulated S_11_ (S_22_) values of − 27.66 dB (− 27.6 dB) for 5G NR mid-band applications, and 5.20–5.46 GHz, with simulated S_11_ (S_22_) values of − 31.28 dB (− 29.47 dB), accommodating 5G NR, Wi-Fi 6, and more wireless standards. The antenna features a straightforward design, including a rectangular patch and a modified ground plane incorporating a semi-elliptical split-ring slot. The individual antenna element has dimensions of 20 mm × 25 mm (0.238λ × 0.298λ at 3.57 GHz), whereas the 2-port antenna measures 50 mm × 25 mm (0.239λ × 0.597λ). Two rectangular strips interconnect both ground planes, facilitating a continuous current distribution. It also aids in reducing undesirable interference among antenna elements, hence decreasing mutual coupling. The interference among antenna elements diminishes by 24 dB in the first band and 20 dB in the second band. The antenna was designed to validate the data acquired using the high-frequency structure simulator (HFSS). The constructed prototype was validated, and the obtained findings exhibit strong concordance with the simulated predictions. The reflection coefficients (S_11_) are less than − 22 dB at 3.50 GHz and − 26.10 dB at 5.35 GHz, although the observed isolation (S_21_) exceeds 22 dB at 3.50 GHz and 20 dB at 5.35 GHz. The antenna exhibits excellent performance in terms of critical performance indicators, including an envelope correlation coefficient (ECC) that does not exceed 0.002 and a diversity gain (DG) of approximately 10 dB. Additionally, the total active reflection coefficient (TARC) is less than − 10 dB, with channel capacity loss (CCL) within the limit of 0.4 bits/sec/Hz and a mean effective gain (MEG) of approximately − 3 dB, which has been evaluated to confirm the suitability of the suggested antenna for diversity and spatial multiplexing features.

**Supplementary Information:**

The online version contains supplementary material available at 10.1038/s41598-026-41860-1.

## Introduction

The antenna is a fundamental component in any communication system. Currently, multi-band and MIMO antennas are required in 5G communication to achieve high gain and low mutual coupling, as they can enhance several performance parameters, including data rate, throughput, stability, and channel capacity^1–3^. MIMO technology makes a significant contribution to the development of 5 G networks. Several antennas are deployed at the sender and receiving sides of the communication system in MIMO technology^4^. The 5G technology is used in several sectors these days, such as agriculture, healthcare, and defence. Without increasing the power required to transmit and/or bandwidth, MIMO technology is the best option to enhance the data rate^5^. As an antenna is an essential part of any communication system, designing compact and efficient antennas is a significant challenge. The MIMO antenna system also has some problems, such as interference between end-to-end placed antenna elements, which degrades the system’s capacity and reliability. Different isolation and interference reduction techniques can be employed to minimise the coupling^6^. Two main reasons primarily cause coupling among different antenna elements: first, from space, which is caused by the near-field radiation of the antenna, and second, the ground side. In this case, the energy from one port to the others is coupled through the ground^7^.

To eliminate the interference between antennas, various techniques have been used, such as a T-shaped neutralisation line in the ground plane^8^, self-decoupling^9,10^ and decoupling strips between antenna elements^11^. In^8^, a T-shaped neutral line was attached to minimise the coupling between two antennas, and two Y-shaped stubs were attached at the other end of the T-shaped structure. However, the isolation is more than 20 dB, but the dimensions of the antenna are 45 mm × 45 mm. A dual-band antenna in^9^ consists of a defective ground plane with a square slot providing isolation of more than 20 dB. No isolation structure is used. Therefore, antennas are self-decoupled; however, designing an antenna with a small size and high isolation is a challenging task. The antenna covering the n78 and n79 bands consists of two semi-circular slots in the patch. The MIMO configuration consists of a square-slotted ground plane with an overall size of 90 mm × 90 mm. It was mentioned that slots were etched in the patch to expand the bandwidth in both bands, as this technique has been a long-standing method to improve bandwidth. Additionally, cross-polarisation reduction is achieved through slots in the patch. Another antenna with a trident-shaped patch and a defective ground was designed in^11^ for the n77/n78/n79 bands, exhibiting a dual-band response.

A rectangular parasitic strip was placed between patches, and a rectangular strip was also added to connect the bottom side of the antenna to decrease the mutual coupling. A gain of 3.14 dBi was attained at two bands. The dimensions of the 2-port antenna were 62 mm × 25.60 mm. A flexible four-element antenna, placed side by side, is described in^12^ for wearable applications in sub-6 GHz bands. A pair of antennas is placed along the x-axis, and then a four-port antenna is achieved by placing two two-element antennas back-to-back. The ground planes are connected using two vertical stubs to minimise mutual coupling. The total size of the final antenna is 62 mm × 52 mm. Several other methods were used to achieve a dual and multi-band response for 5G applications operating in different frequency bands while maintaining sufficient mutual coupling by employing various isolation structures, as discussed in^12–18^. Most of these antenna designs discussed in the literature are either large or employ a complex design structure.

The significant contribution of this paper is summarised below:

1. The two-port antenna’s electrical dimensions are 0.239λ × 0.597λ, which is less than those of the other reported antennas. In terms of the unit area, the proposed antenna is smaller than the 2-port and 4-port antennas.
2. It operates at two different frequencies: 3.50–3.68 GHz and 5.20–5.46 GHz, making it relevant for multiple 5G NR (n48, n77, n78, n46), Wi-Fi 6, and other wireless services.
3. The antenna is simple in structure without any isolation structure; the isolation between antenna elements exceeds 24 dB and 20 dB within two operating bands. Consequently, the antenna’s complexity is minimal.
4. Shows excellent MIMO parameters, which include ECC under 0.002, DG close to 10 dB, CCL less than 0.4 bps/Hz and MEG of 3 dB.

In this paper, we have designed a dual-band two-port antenna by placing two single antenna elements parallel to the x-axis for 5G applications. It operates in the 3.50–3.68 GHz and 5.20–5.46 GHz bands, which are suitable for 5G bands n46 (5.15–5.925 GHz), n48 (3.55–3.7 GHz), n77 (3.3–4.2 GHz), n78 (3.3–3.8 GHz) and LTE band n42 (3.4–3.6 GHz). The antenna is simple in structure, featuring a patch and a defective ground plane with a semi-elliptical and split-ring slot. HFSS is used to simulate the design, and different parameters are investigated. Both ground planes are connected using two horizontal rectangular stubs to achieve an isolation of more than − 20 dB (Table 1).

**Table 1:** Different frequency bands of 5G in the sub-6 GHz region.

Designated band	Operating frequency
n42	3.4–3.6 GHz
n46	5.15–5.925 GHz
n48	3.55–3.7 GHz
n77	3.3–4.2 GHz
n78	3.3–3.8 GHz

## Antenna geometry

### Geometry of the single antenna

**Fig. 1:**
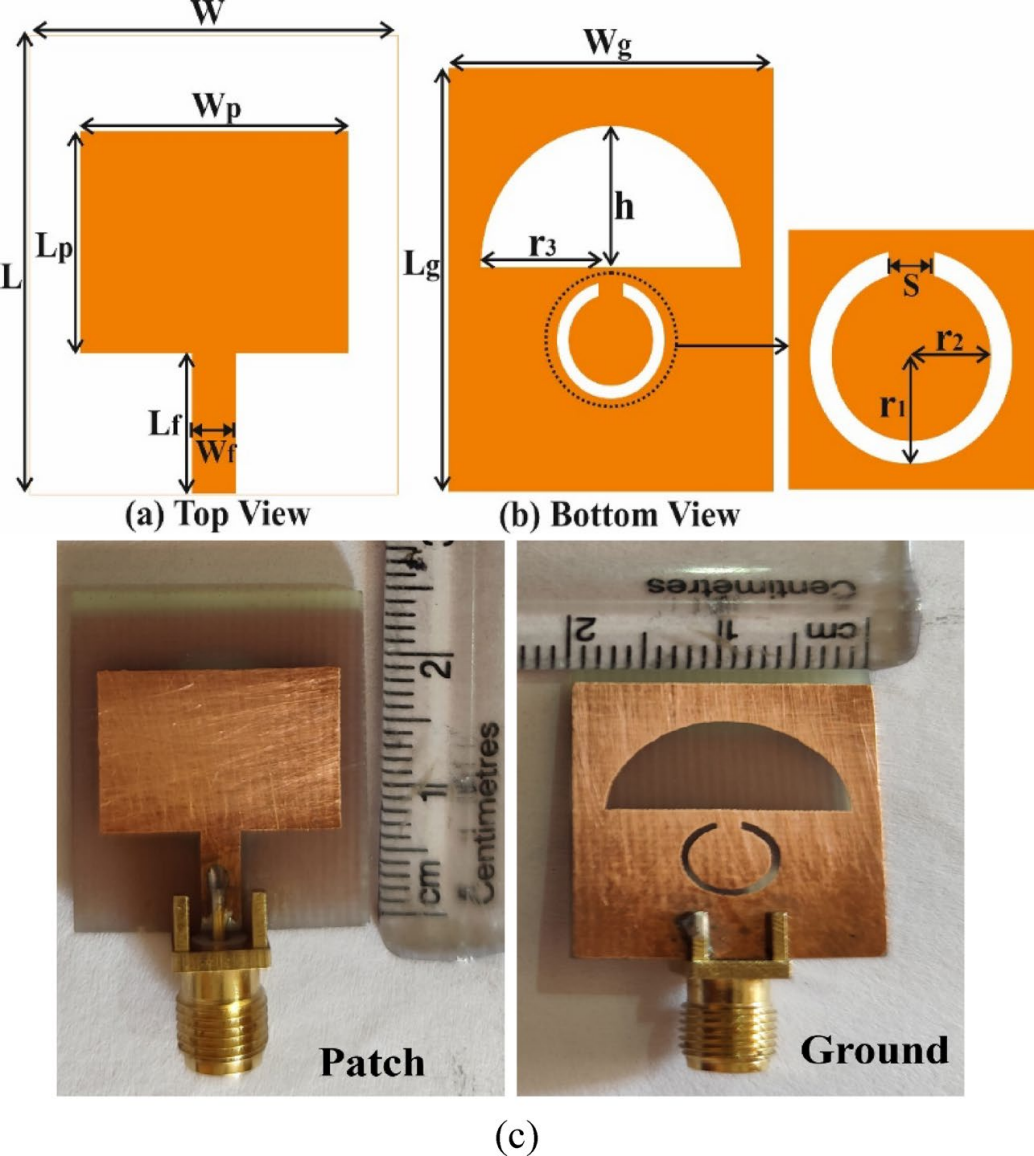
Geometry of the designed Antenna, where (**a**) shows the top view, (**b**) shows the bottom view, and (**c**) presents the fabricated prototype of the proposed antenna.

The design structure of the presented antenna, with patch and bottom views, is depicted in Fig. 1a and b, respectively. The dimensions of the single antenna are 20 mm × 25 mm. The antenna is implemented on FR-4, having a thickness of 1.6 mm, a dielectric constant of 4.4, and a loss tangent of 0.02^19^.

The final antenna involves a patch with a rectangular shape and a defective ground plane. The microstrip feed line is used to feed the RF signal to the antenna. The bottom side is defective, featuring a semi-elliptical space with a significant radius of r_3_ and a minor radius of h, along with a split ring slot. The feed line width is fixed at 2.7 mm. The length Lg is reduced to 24 mm to achieve perfect matching with a 50 Ω feed line at the specified operating frequencies. Additionally, the position of the slots in the ground is optimised for the desired operating frequencies.

**Fig. 2:**
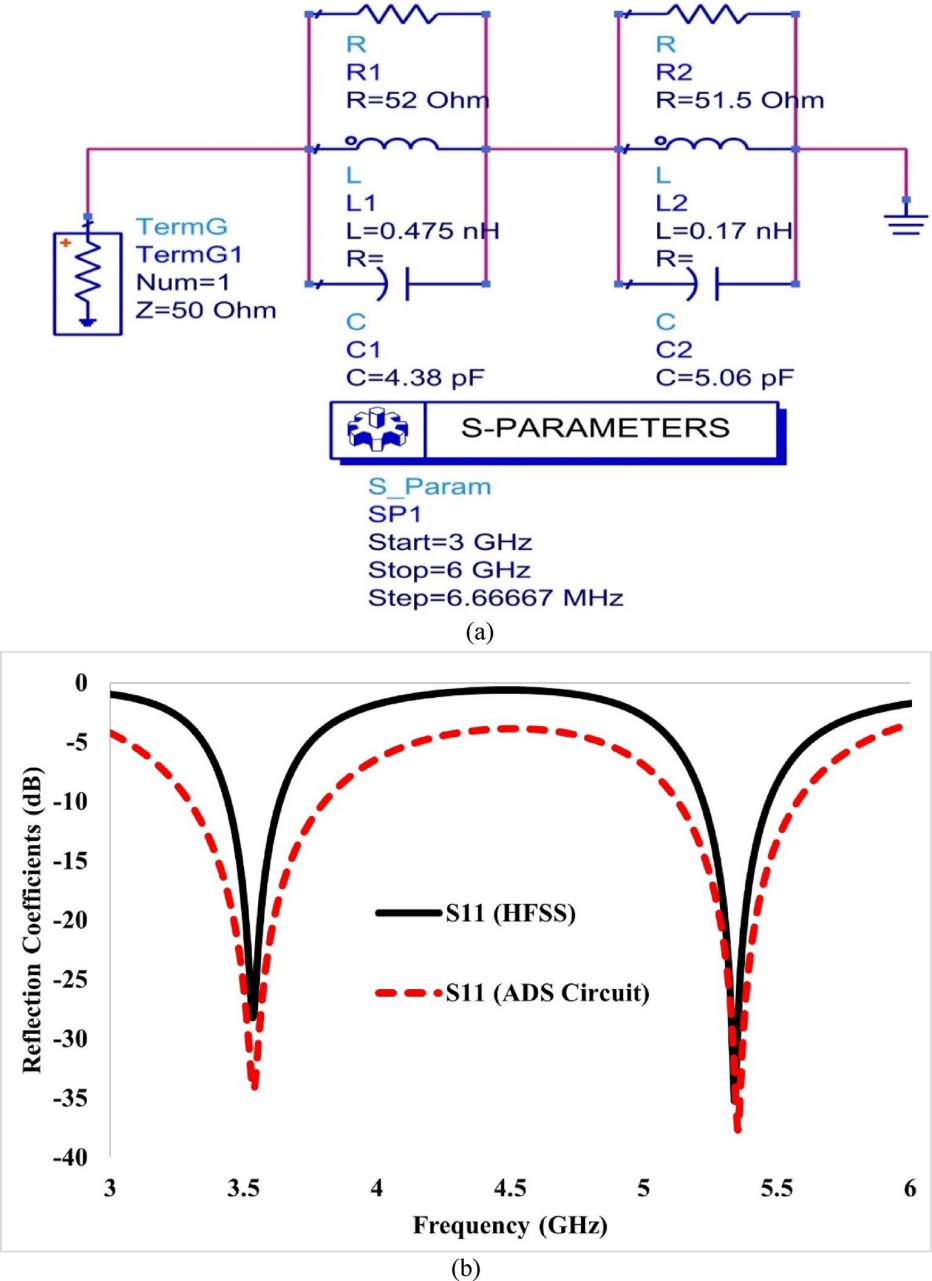
(**a**) Equivalent circuit model and (**b**) comparison of predicted S-parameters from HFSS and the equivalent circuit for the single antenna.

The final design parameters of the proposed structure are summarised in Table 2, and the predicted return loss parameters from HFSS of the suggested single antenna element are displayed in Fig. 2. The developed antenna can be represented as an equivalent electronic circuit. Generally, the equivalent circuit model of an antenna is preferred because an accurate simulation of the antenna in an EM simulator often consists of complex configurations^20^. To design the equivalent circuit of a two-port antenna, the first step is to design the equivalent circuit of a single antenna element with a dual-band response, utilising the Advanced Design System (ADS) software from Keysight Technologies^21^.

**Table 2:** Dimensional parameters of antenna element.

Parameter	Value (mm)	Parameter	Value (mm)
L	25	L_g_	24
W = W_g_	20	S	1.5
L_p_	12	r_1_	3.3
W_p_	16.5	r_2_	2.6
L_f_	7.6	r_3_	8
W_f_	2.7	h	7.75

This equivalent circuit, as a series combination of two parallel RLC circuits, is shown in Fig. 2a. The first RLC circuit (R_1_L_1_C_1_) controls the first operating frequency (3.57 GHz) while the circuit R_2_L_2_C_2_ controls the second operating frequency. Both the resonant frequencies of the developed antenna are calculated using Equations by following the process as given in^22^. The impedance of the first parallel circuit is given by Eq. (1).

1 $$\begin{aligned} \frac{1}{{Z_{1} }} = & \, \frac{1}{{R_{1} }} + \frac{1}{{j\omega L_{1} }} + j\omega C_{1} = \frac{1}{{R_{1} }} - \frac{j}{{\omega L_{1} }} + j\omega C_{1} \\ \frac{1}{{Z_{1} }} = & \, \frac{{\omega L_{1} + j\left( {\omega^{2} L_{1} C_{1} R_{1} - R_{1} } \right)}}{{\omega L_{1} R_{1} }} \\ Z_{1} = & \,\frac{{\omega L_{1} R_{1} }}{{\omega L_{1} + j\left( {\omega^{2} L_{1} C_{1} R_{1} - R_{1} } \right)}} \\ Z_{1} = & \,\frac{{\omega L_{1} R_{1} \left[ {\omega L_{1} - j\left( {\omega^{2} L_{1} C_{1} R_{1} - R_{1} } \right)} \right]}}{{\left[ {\omega L_{1} + j\left( {\omega^{2} L_{1} C_{1} R_{1} - R_{1} } \right)} \right]\left[ {\omega L_{1} - j\left( {\omega^{2} L_{1} C_{1} R_{1} - R_{1} } \right)} \right]}} \\ Z_{1} = & \,\frac{{\omega^{2} L_{1}^{2} R_{1} }}{{\omega^{2} L_{1}^{2} + \left( {\omega^{2} L_{1} C_{1} R_{1} - R_{1} } \right)^{2} }} + j\frac{{\omega L_{1} R_{1}^{2} - \omega^{3} L_{1}^{2} C_{1} R_{1}^{2} }}{{\omega^{2} L_{1}^{2} + \left( {\omega^{2} L_{1} C_{1} R_{1} - R_{1} } \right)^{2} }} \\ \end{aligned}$$

The imaginary part of the Z_1_ is

$$Z_{1, imaginary} = \frac{{\omega L_{1} R_{1}^{2} - \omega^{3} L_{1}^{2} C_{1} R_{1}^{2} }}{{\omega^{2} L_{1}^{2} + \left( {\omega^{2} L_{1} C_{1} R_{1} - R_{1} } \right)^{2} }}$$

To calculate the first resonant frequency, put the imaginary part of Z_1_ equal to zero.

2 $$\begin{aligned} & Z_{1, imaginary} = 0 \\ & \frac{{\omega L_{1} R_{1}^{2} - \omega^{3} L_{1}^{2} C_{1} R_{1}^{2} }}{{\omega^{2} L_{1}^{2} + \left( {\omega^{2} L_{1} C_{1} R_{1} - R_{1} } \right)^{2} }} = 0 \\ & \omega L_{1} R_{1}^{2} - \omega^{3} L_{1}^{2} C_{1} R_{1}^{2} = 0 \\ & \omega L_{1} R_{1}^{2} \left( {1 - \omega^{2} L_{1} C_{1} } \right) = 0 \\ \end{aligned}$$

Solving Eq. (2), the first resonance frequency of the circuit is calculated using Eq. (3).

3 $$\begin{aligned} f_{1} = & \,\frac{1}{{2\pi \sqrt {L_{1} C_{1} } }} \\ f_{1} = & \,\frac{1}{{2\pi \sqrt {\left( {0.475 \times 10^{ - 9} } \right)\left( {4.38 \times 10^{ - 12} } \right)} }} = {3}.{\text{48 GHz}} \\ \end{aligned}$$

Similarly, the second resonance frequency of the antenna is determined using Eq. (4).

4 $$\begin{aligned} f_{2} = & \,\frac{1}{{2\pi \sqrt {L_{2} C_{2} } }} \\ f_{2} = & \,\frac{1}{{2\pi \sqrt {\left( {0.17 \times 10^{ - 9} } \right)\left( {5.06 \times 10^{ - 12} } \right)} }} = {5}.{\text{43 GHz}} \\ \end{aligned}$$

The frequencies calculated from the equivalent circuit’s lumped elements are 3.48 and 5.43 GHz, which are close to the frequencies obtained from HFSS, which are 3.57 and 5.35 GHz. The S-parameters for the single antenna, computed using the HFSS and ADS simulators, are plotted in Fig. 2b.

Figure 2b shows that the reflection coefficients from the HFSS and ADS simulations are in close agreement, thereby validating the model’s effectiveness in representing the developed antenna at the specified frequencies. Table 3 summarises the optimised values of the elements used in the equivalent circuit model.

**Table 3:** Optimal values of circuit components.

Resistance (Ω)	Capacitance (pF)	Inductance (nH)
R_1_ = 52	C_1_ = 4.38	L_1_ = 0.475
R_2_ = 51.5	C_2_ = 5.06	L_2_ = 0.170

### Antenna design procedure

**Fig. 3:**
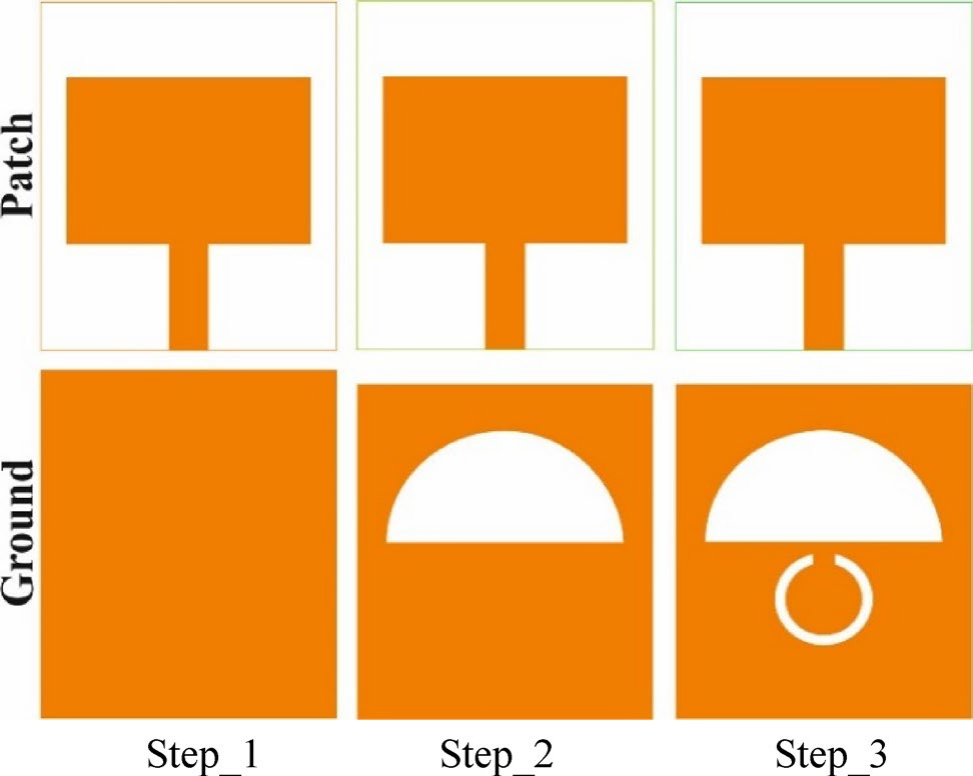
Design Steps followed to achieve the suggested antenna.

The stepwise evolution to obtain the final antenna design has been executed to achieve a dual-band response. The various stages of design evolution are illustrated in Fig. 3. The initial antenna design (step 1) originated from a simple rectangular patch with a full ground plane, and the Eqs. (5)–(8) were used to find the dimensions of the patch and the initial resonance frequency.

Initially, we selected 5.6 GHz as the resonance frequency (f_0_). So, for FR-4 substrate with ε_r_ = 4.4 and a thickness of 1.6 mm, the width of the patch is given by Eq. (5)^23^.

5 $$W = \frac{c}{{2f_{0} \sqrt {\frac{{\varepsilon_{r} + 1}}{2}} }}$$

where c is the speed of EM waves in free space, for a frequency of 5.6 GHz, the width of the patch is calculated to be 16.28 mm and is set to be 16.5 mm.

The effective length of the patch can be calculated using Eq. (6)^23^.

6 $$L_{e} = \frac{c}{{2f_{0} \sqrt {\varepsilon_{e} } }} = L + \Delta L$$

ε_e_ is the effective dielectric constant, L is the physical length of the patch, and ΔL is the fringing length.

From Eq. (6), for a frequency of 5.6 GHz, the effective length of the patch is calculated to be 13.54 and the fringing length ΔL is given by Eq. (7)^23^.

7 $$\Delta L = \frac{h}{{\sqrt {\varepsilon_{e} } }} = 0.812\,{\text{ mm}}$$

So, the physical length L of the patch is

$${\mathrm{L}} = {13}.{54} - {2} \times 0.{812} = {11}.{92}\,{\mathrm{mm}}$$

Since the calculated value of L is 11.92 mm, and we have selected the patch length as 12 mm for the suggested antenna. The resonance frequency (f_0_) of the antenna excited at any TM_mn_ mode can be obtained using Eq. (8) ^24^.

8 $$f_{0} = \frac{c}{{2\sqrt {\varepsilon_{e} } }}\left[ {\left( {\frac{m}{{L_{e} }}} \right)^{2} + \left( \frac{n}{W} \right)^{2} } \right]^{\frac{1}{2}}$$

**Fig. 4:**
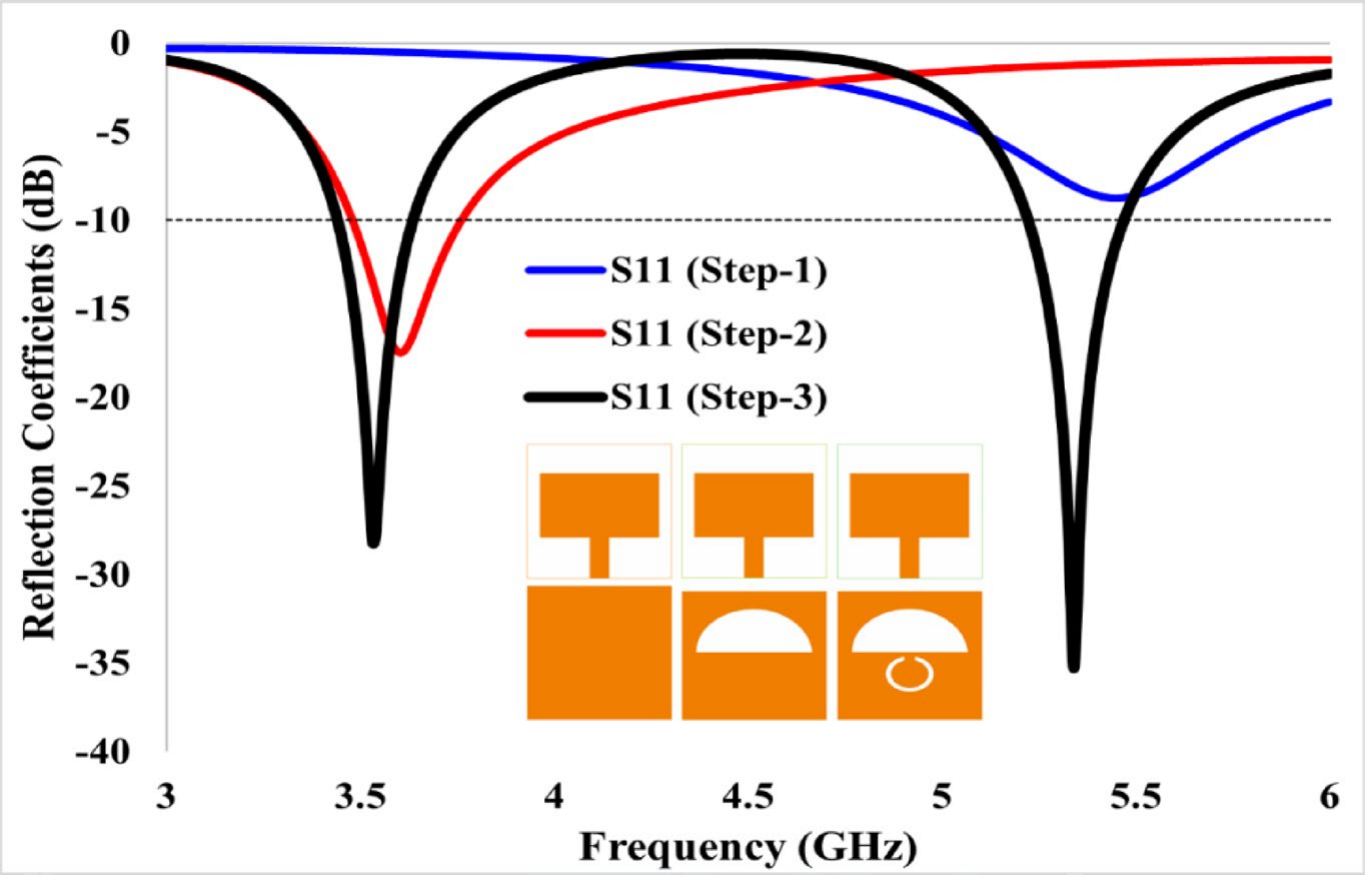
S-parameters of various design steps, illustrating the evolution of return loss during the antenna design process.

Using Eq. (8), for the TM_10_ (m = 1, *n* = 0) mode, the resonance frequency obtained is 5.59 GHz; however, it fails to achieve satisfactory performance in terms of impedance matching, as evident from Fig. 4.

In the next stage (step 2), the ground plane is modified with a semi-elliptical slot, enabling single-band operation at 3.60 GHz with improved impedance matching.

Also, the length L_g_ is set to 24 mm. Finally, to achieve another band of operation (5.20–5.46 GHz), a circular split-ring slot is created within the ground (step 3). However, the antenna does not operate at the desired frequencies, so some of the parameters were adjusted.

Since the structure in step 3 makes the antenna operate at 5.32 GHz and achieve the desired dual-band operation, it is deemed to be the final antenna design. The return loss parameters obtained from the simulation corresponding to different design stages are shown in Fig. 4.

#### Parametric analysis

**Fig. 5:**
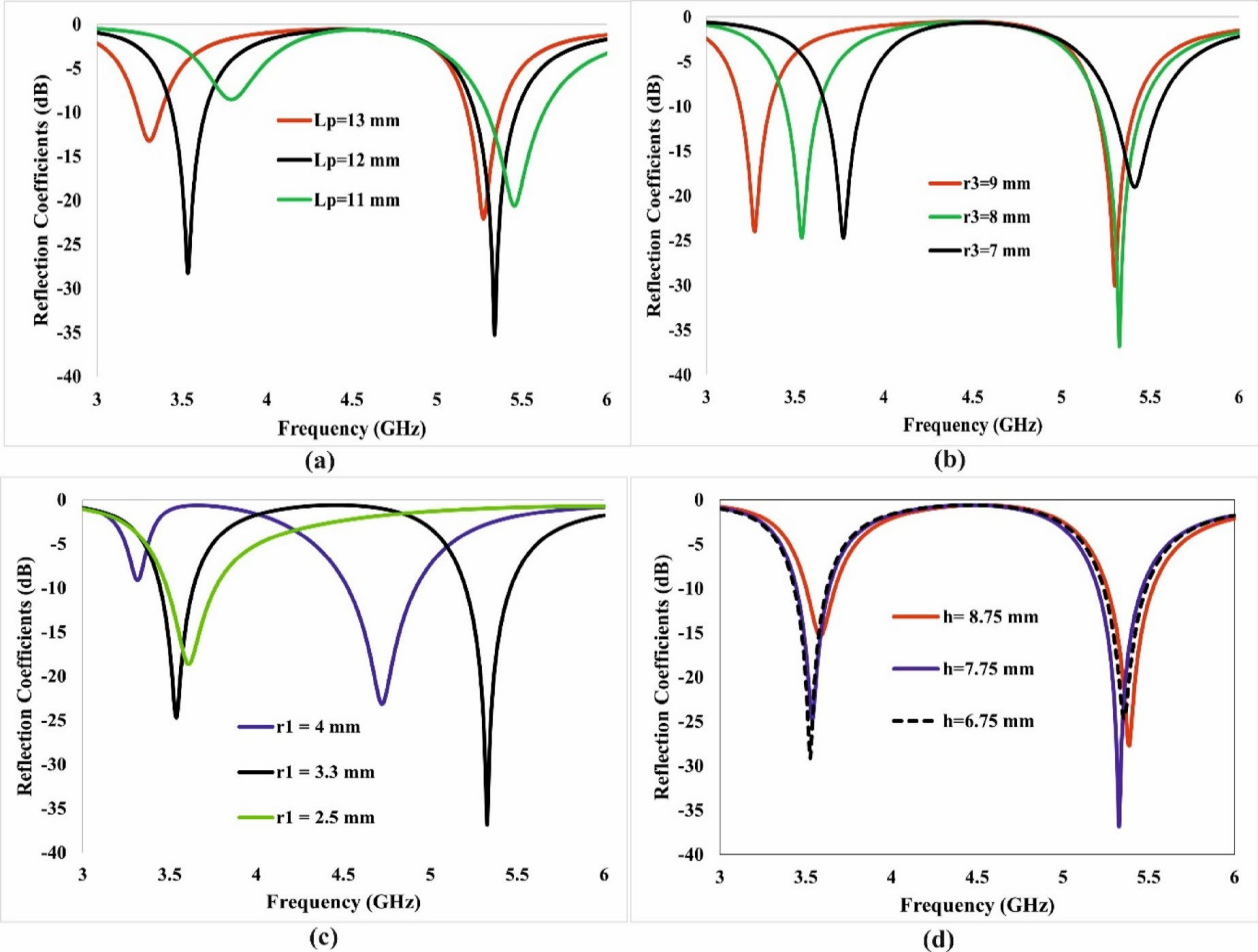
Parametric analysis of the proposed antenna for (**a**) L_p,_ (**b**) r_3,_ (**c**) r_1,_ (**d**) h, showing their influence on the impedance matching and operating frequency.

The impact of varying different design parameters on antenna performance, in terms of operational frequency and reflection coefficients, is examined. These parameters include L_p_, r_3_, r_1,_ and h. The S-parameters of this parametric analysis are presented in Fig. 5. This variation in different parameters demonstrates that it is possible to alter the impedance matching, and the operating frequency can be shifted as required. Figure 5a indicates that the variation in L_p_ strongly impacts impedance matching of the lower band, but a slight shift in frequency of the upper band is observed. It is shown that increasing the length of the patch (Lp) beyond 12 mm results in a slight shift in the operating frequency of the upper band to the left and right, with a reduction in Lp. Also, the frequency and impedance matching of the lower band changed with L_p_. The ideal value of L_p_ is 12 mm. Similarly, by increasing the radius of the elliptical slot (r_3_), the targeted frequency of the first band can be shifted to the left.

In contrast, the targeted frequency can be shifted to the right by decreasing the radius r_3_. The upper band remains almost unaffected in terms of operating frequency. Modification in r_3_ has an impact on the reflection coefficient behaviour of the antenna in the upper band, but it controls the reflection coefficient of the second band. Therefore, the value of r3 is set to 8 mm to achieve the desired operating frequency in the lower band, as well as good impedance matching in the upper band.

The variation in the outer radius of the split ring (r_1_) significantly influences the working frequency as well as the S-parameters of the Antenna in the first band. An increase in r_1_ shifts the upper-band frequency toward the lower side; however, this adjustment causes the antenna to lose its dual-band functionality. Conversely, reducing r_1_ restricts the antenna’s operation to the lower band only. To ensure dual-band operation, r_1_ is optimised at 3.3 mm, as depicted in Fig. 5c.

Furthermore, Fig. 5d illustrates the impact of varying h from 6.75 mm to 8.75 mm on the antenna’s performance. Adjusting the parameter h has a negligible effect on the operating frequencies of both bands but plays a crucial role in optimising impedance matching. The variation in h does not significantly change the operating frequency of either band, but it affects impedance matching in both bands.

To ensure better impedance matching at the target frequencies, h is set to 7.75 mm, as per Fig. 5d.

#### Surface current distribution

**Fig. 6:**
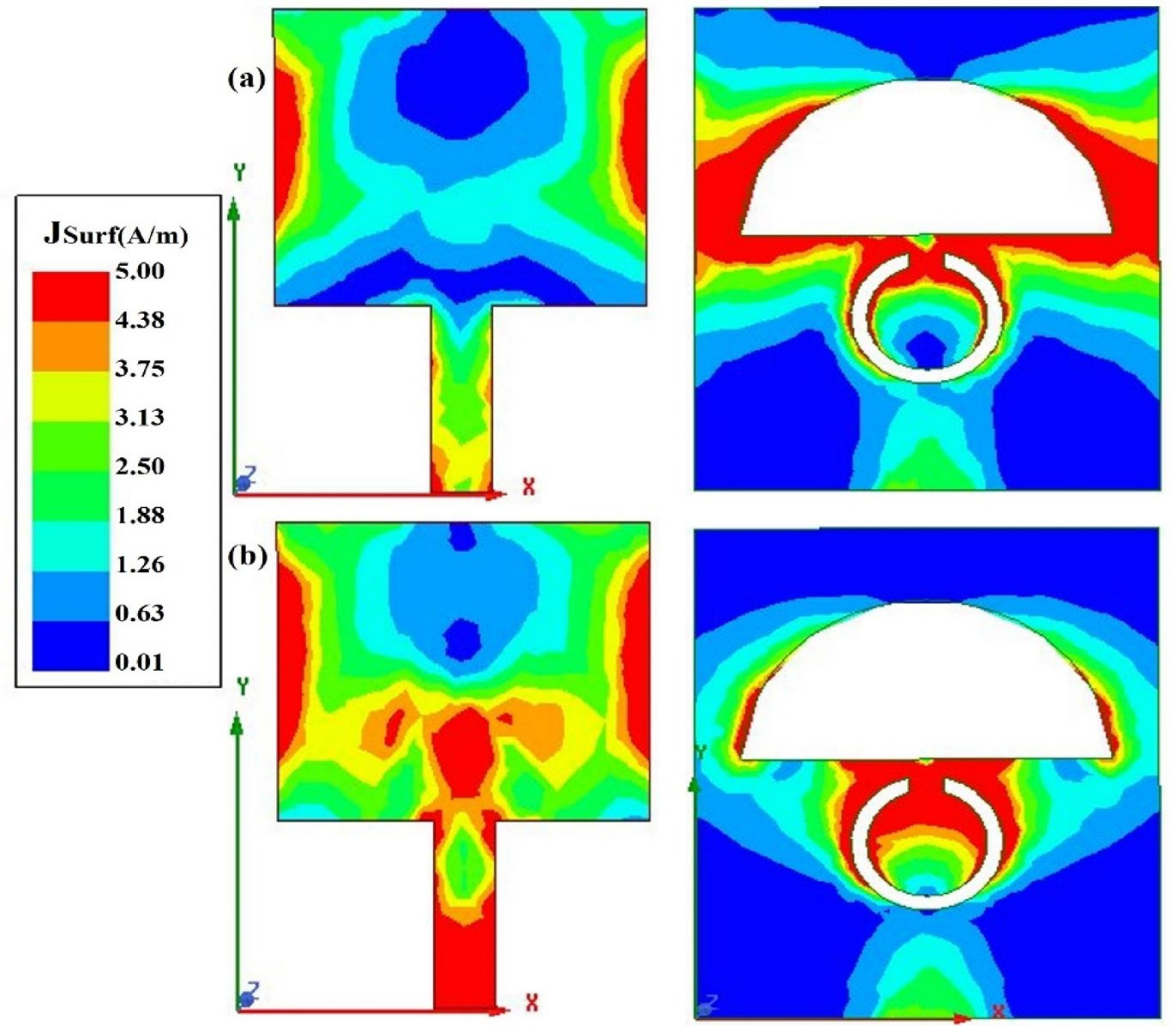
Surface current distribution of the proposed antenna at (**a**) 3.57 GHz and (**b**) 5.32 GHz, illustrating the current flow responsible for resonance at both frequencies.

Figure 6a and b show the simulated surface current at 3.54 GHz and 5.32 GHz, which are the two resonant frequencies. From Fig. 6a, it can be understood that most of the current is distributed at the boundaries of the patch and the semi-circular slot in the ground. A small amount of current is also concentrated at the upper portion of the split ring slot at 3.54 GHz. At 5.32 GHz, the current is distributed evenly between the top radiator and the feedline. Also, the highest current is distributed at the upper edges of the split ring slot in the ground plane.

#### Results of single antenna element

**Fig. 7:**
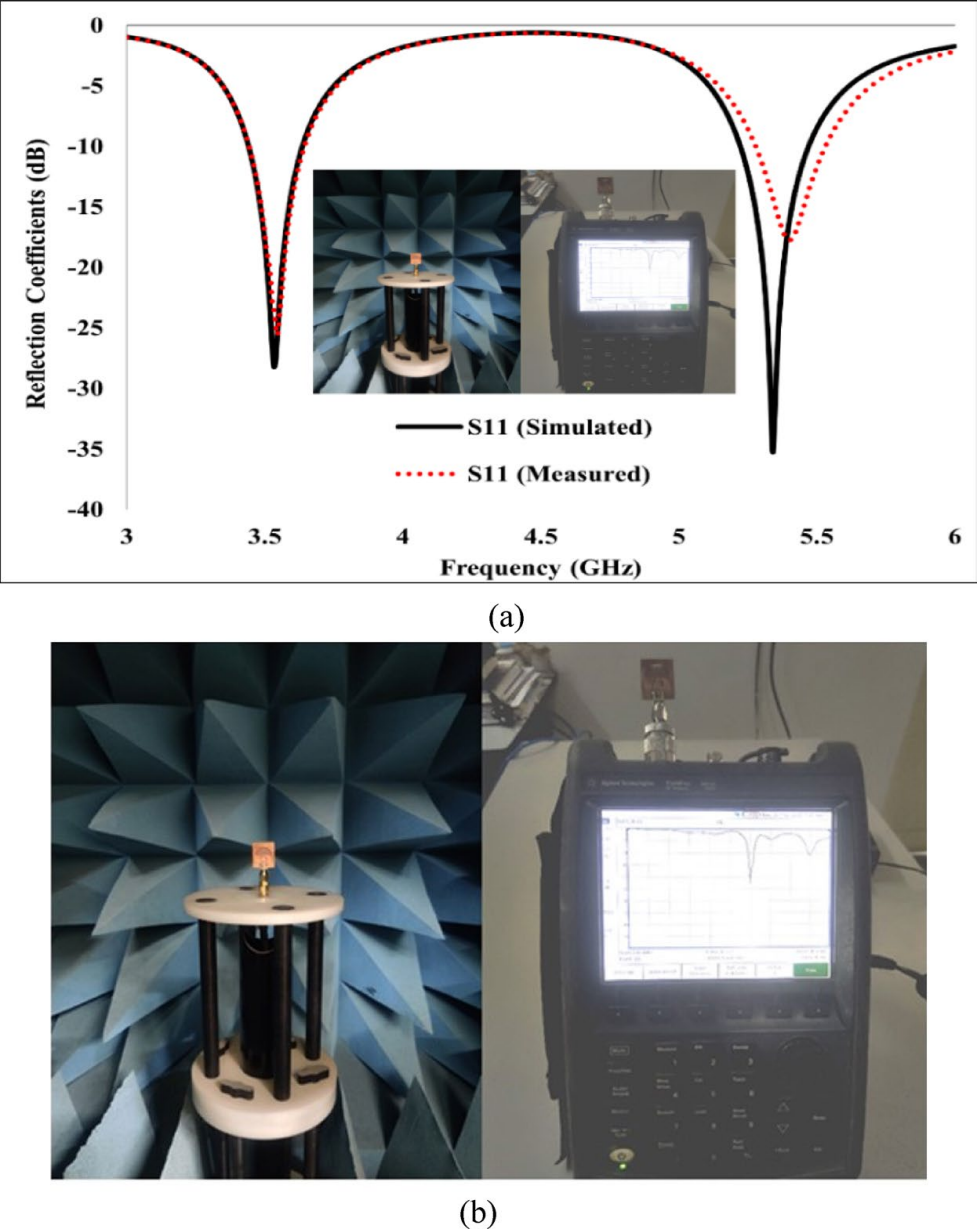
(**a**) S-Parameters of single antenna element, (**b**) Measurement Setup of Single Antenna.

FR-4 substrate was used to fabricate the proposed design due to its low cost and widespread availability. Figure 7a illustrates a prototype of the suggested antenna, whilst the measurement configuration and return loss parameters are depicted in Fig. 7b and c, respectively. Figure 7a demonstrates that the simulated and experimental S-parameters align precisely, except for a slight discrepancy in S11 at the upper band.

## 2-port MIMO antenna

### Geometry

**Fig. 8:**
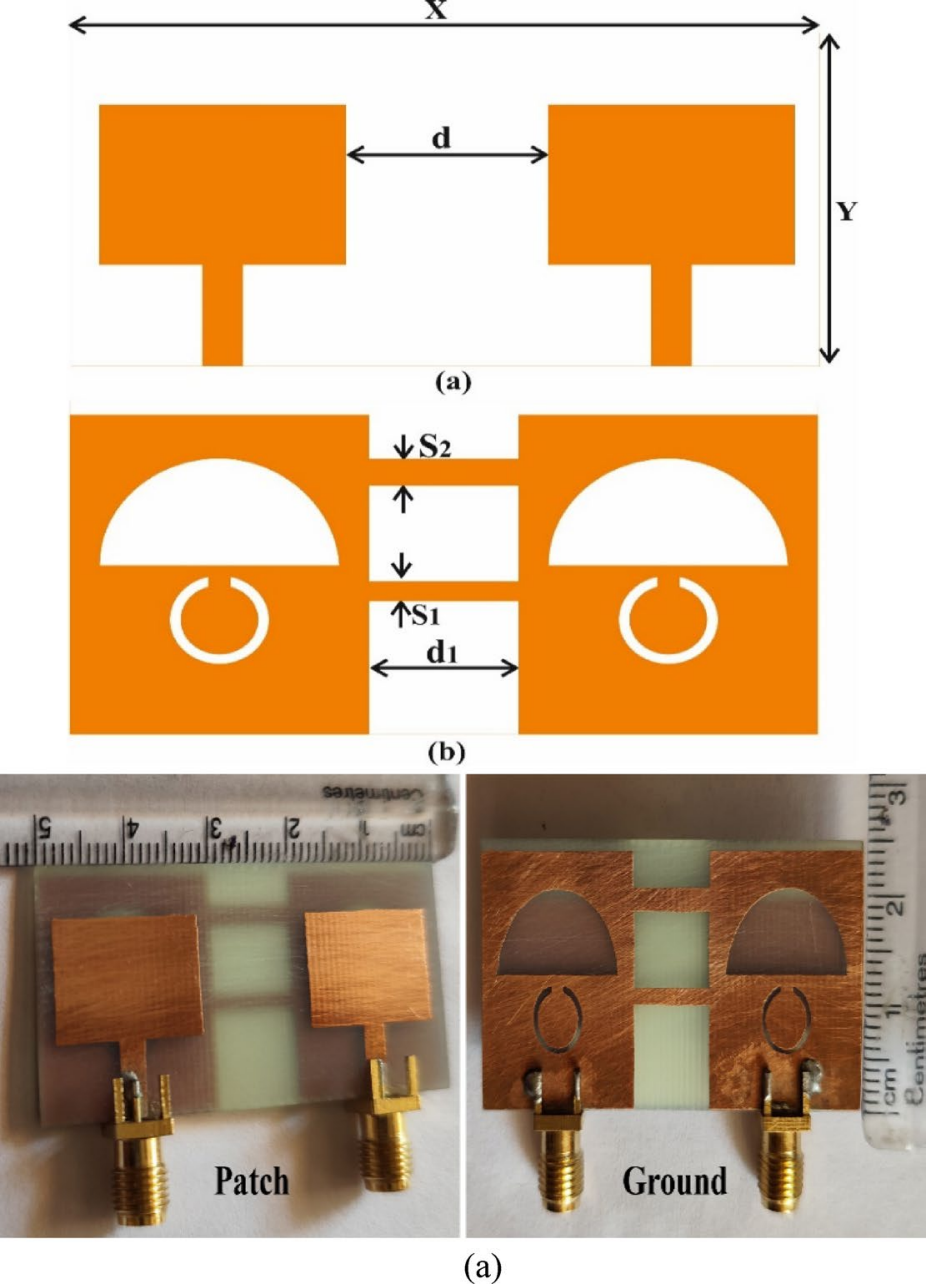
Design geometry of 2-port antenna (**a**) patch side (**b**) ground side (**c**) prototype of the fabricated antenna.

To realise a two-port MIMO antenna, two identical single antenna elements are symmetrically positioned along the horizontal axis with an edge-to-edge spacing of d. The ground planes of both elements are interconnected using two rectangular decoupling strips, each with dimensions d1 × S1 and d1 × S2, respectively. Figure 8c presents the fabricated 2-port antenna, which has overall dimensions of 50 mm × 25 mm. Its key design parameters are summarised in Table 4.

**Table 4:** Design Parameters of the Two-port Antenna.

Parameter	Value (mm)
X	50
Y	25
D	13.5
d_1_	10
S_1_	1.5
S_2_	1.9

**Fig. 9:**
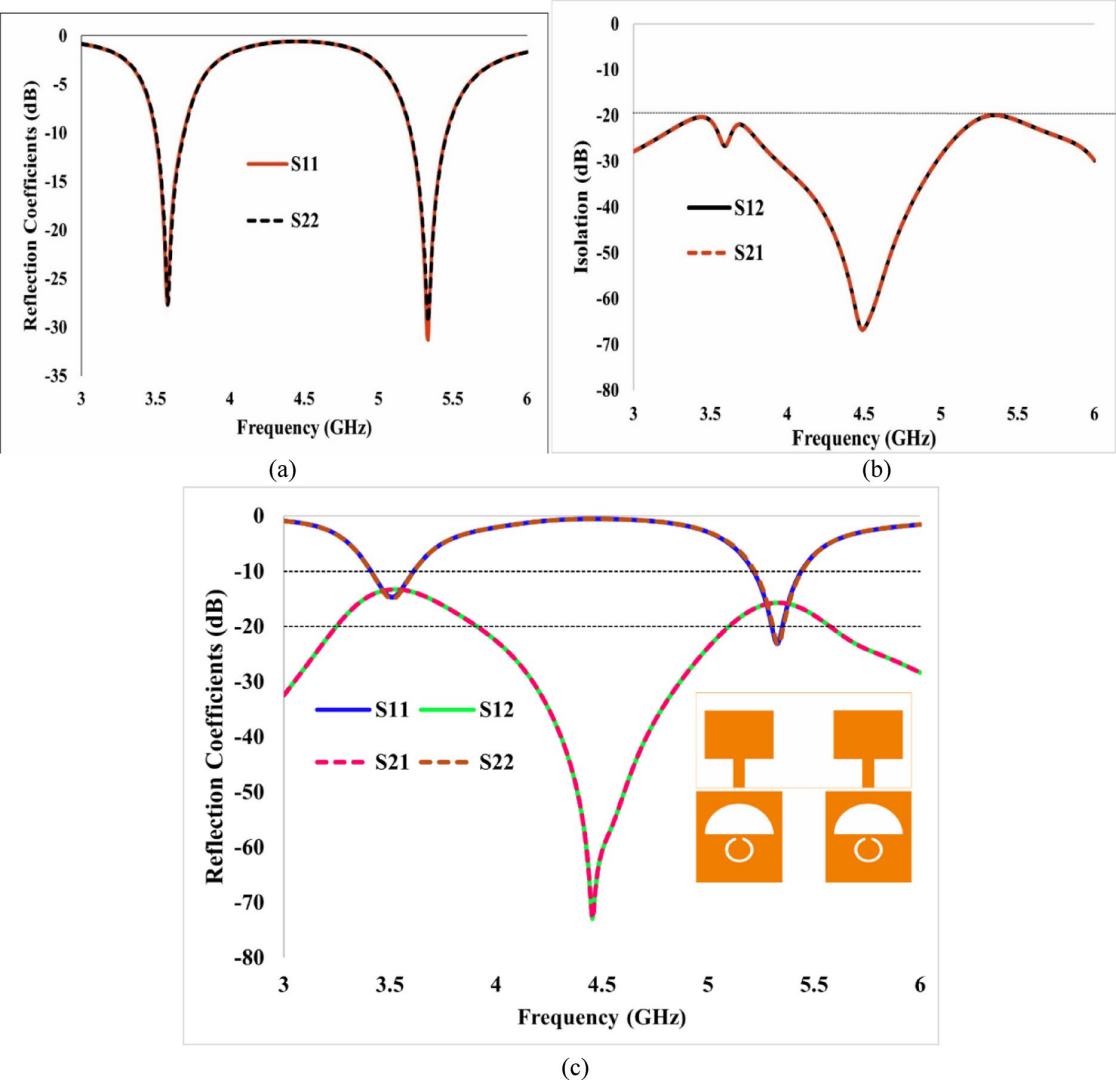
Simulated S-parameters of the proposed two-port Antenna: (**a**) Reflection coefficients illustrating the dual-band response with good impedance matching, (**b**) Transmission coefficients with connected ground planes demonstrating strong isolation between the ports, and (**c**) S-parameters without connected ground planes.

**Fig. 10:**
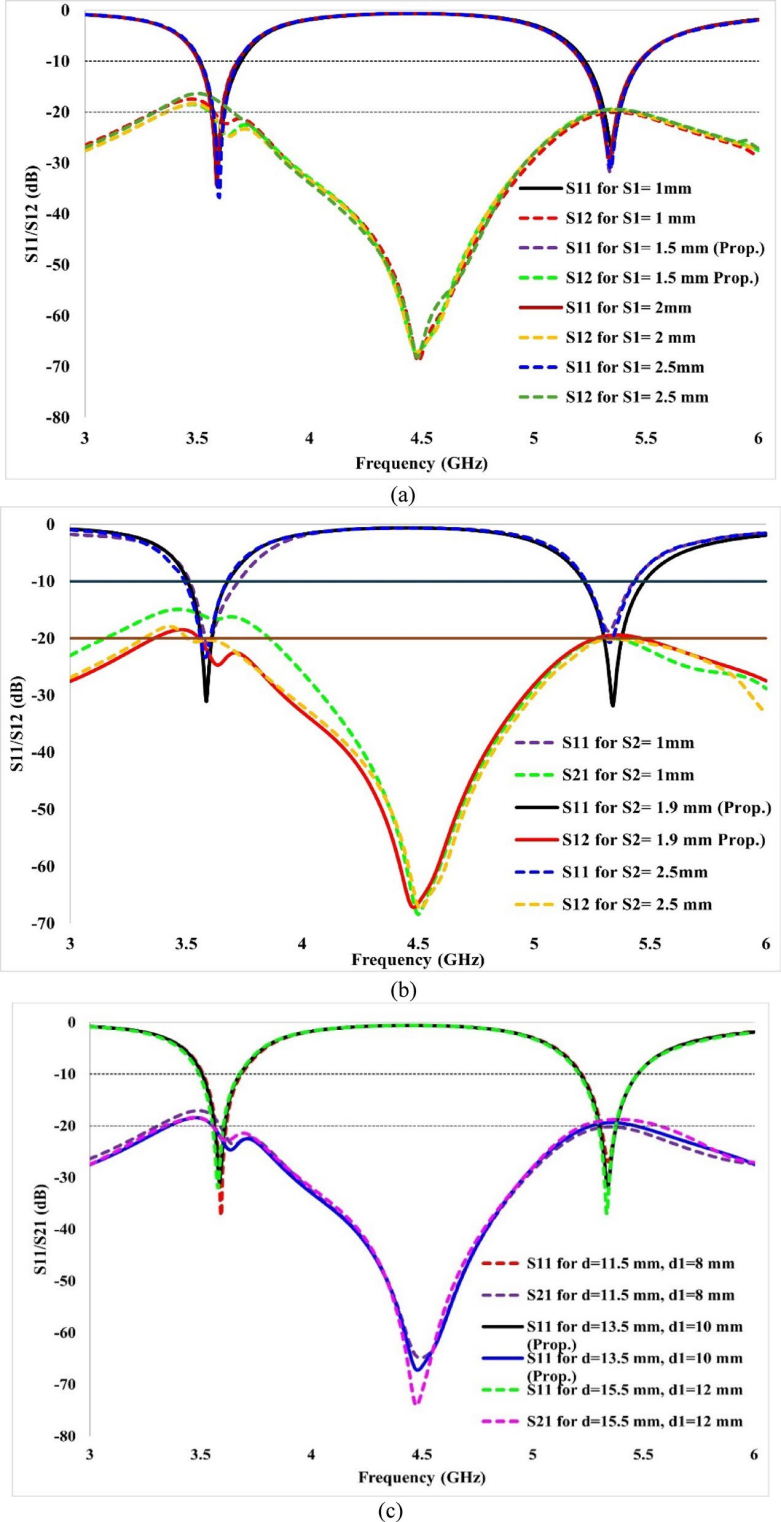

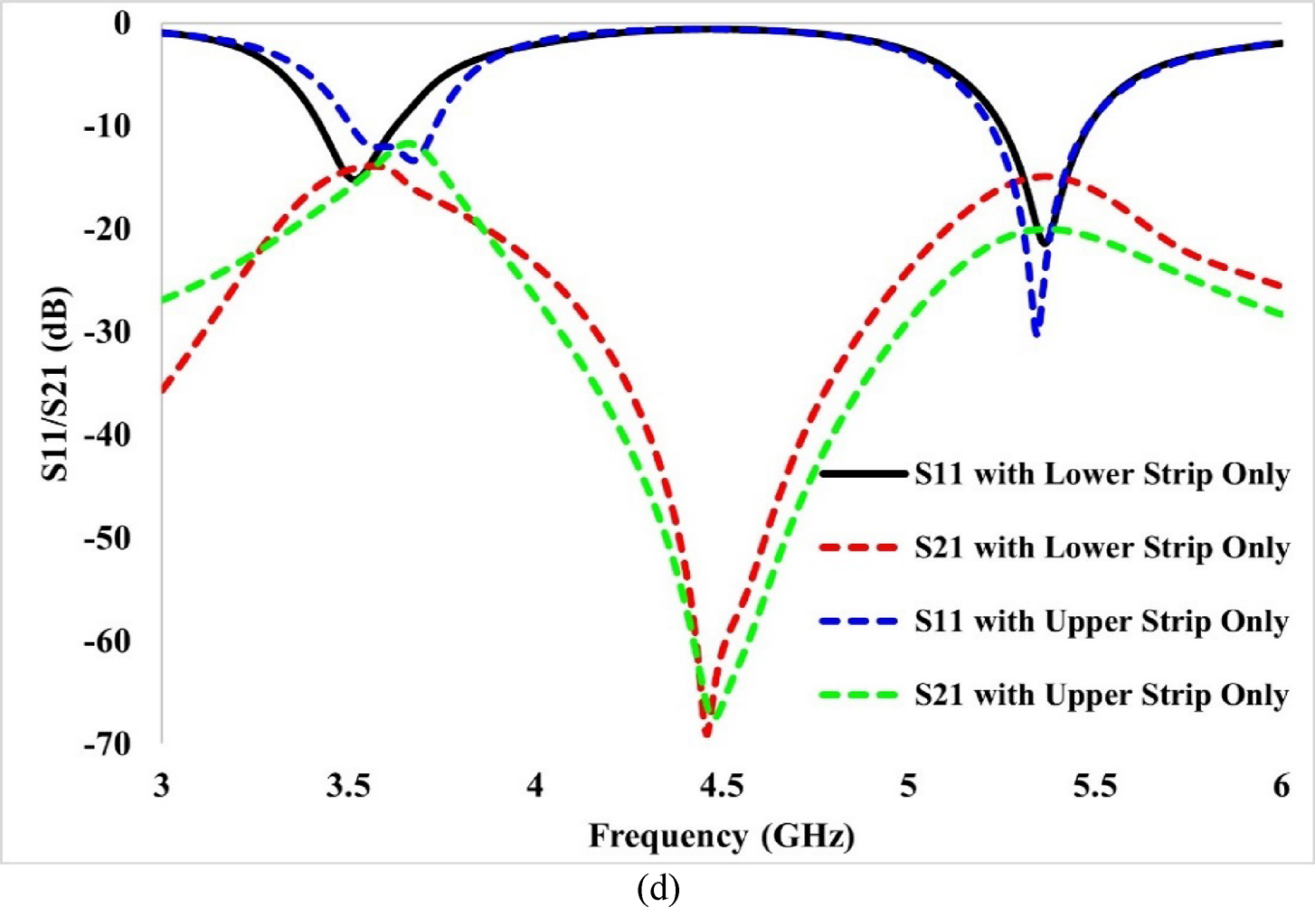
Parametric analysis of 2 port antenna for (**a**) S_1,_ (**b**) S_2,_ and (**c**) d and d_1_ (**d**) only one decoupling strip is used.

The simulated return loss and isolation characteristics for this configuration are illustrated in Fig. 9a and b. The effective isolation is ensured by maintaining optimal inter-element spacing and employing decoupling strips to bridge the ground planes. The spacing between the ground planes cannot be increased, as it will increase the antenna size. In contrast, Fig. 10 presents the return loss and isolation performance without connecting ground planes, demonstrating inadequate isolation levels.

Also, if the grounds of individual antennas remain unconnected, the practicality of the design for real-world MIMO applications is compromised, as a common reference plane is essential for maintaining stable performance without degrading antenna characteristics^25^.

The proposed design achieves an isolation of more than − 20 dB across both operating frequency bands by connecting the ground planes, thereby enhancing MIMO system performance.

Figure 9 presents the simulated return loss and isolation characteristics, demonstrating isolation across the operating frequencies of both bands.

To observe the impact of the dimensions of decoupling strips between the ground plane and their spacing, analysis has also been conducted. To effectively isolate both antenna elements, it is essential to optimise the dimensions of decoupling strips and spacing between them. So, we have performed a series of parametric studies and the dimensions and position of decoupling structures were optimised. The length, width, and their position or spacing between them were varied, and their impact on S-parameters was analysed. So, final dimensions and spacing between strips were selected to achieve better isolation with adequate impedance matching and other performances.

For this, we have performed a parametric analysis for d, d_1_, S_1_ and S_2_. The impact of these four parameters on mutual coupling is illustrated in Fig. 10a–c. As per Fig. 10a, increasing S_1_ affects the mutual coupling across the lower band. It has no significant effect on the reflection coefficient. Increasing the S_1_, the isolation between antenna elements decreases. Similarly, S_2_ plays a crucial role in increasing isolation. Decreasing the S_2_ reduces the isolation across the lower band. It also has an impact on reflection coefficients across both the operating bands. Increasing the S_2_ beyond its optimal value reduces the reflection coefficients. It can be observed from Fig. 10b. The edge-to-edge spacing between the patch and ground planes is also an important parameter. The parameters ‘d’ denotes the spacing between the patches of two antennas, while ‘d_1_’ denotes the spacing between the ground planes. These two parameters were varied simultaneously, as it is not possible to vary them independently, and this approach is also not physically meaningful. Their effect on impedance matching, resonant frequency and isolation was studied, and their optimised values were identified.

Figure 10c illustrates the impact of varying these parameters. Decreasing the values of distance reduces the isolation across the upper band (5.32 GHz). It does not affect the isolation in the lower band, but it reduces the reflection coefficients. Increasing the spacing between antennas reduces the isolation across the second band (5.32 GHz) and has a slight effect on the isolation across the lower band. It has no significant role in influencing the S_11_.

A study has also been conducted on the role of using two decoupling strips. It is presented in Fig. 10d. From Fig. 10, it can be observed that with the use of one strip, the antenna exhibits poor reflection coefficients and inadequate isolation performance. When either the lower or upper strip is placed individually, the antenna performance deteriorates—using only the lower strip results in poor impedance matching across both operating bands, with the isolation level limited to about 15 dB, which is inadequate. In contrast, with only the upper strip, the impedance matching remains acceptable at the upper frequency band but degrades at the lower band. Although the isolation is unaffected at the upper band, it falls below 12 dB at the lower band, indicating insufficient isolation performance.

**Fig. 11:**
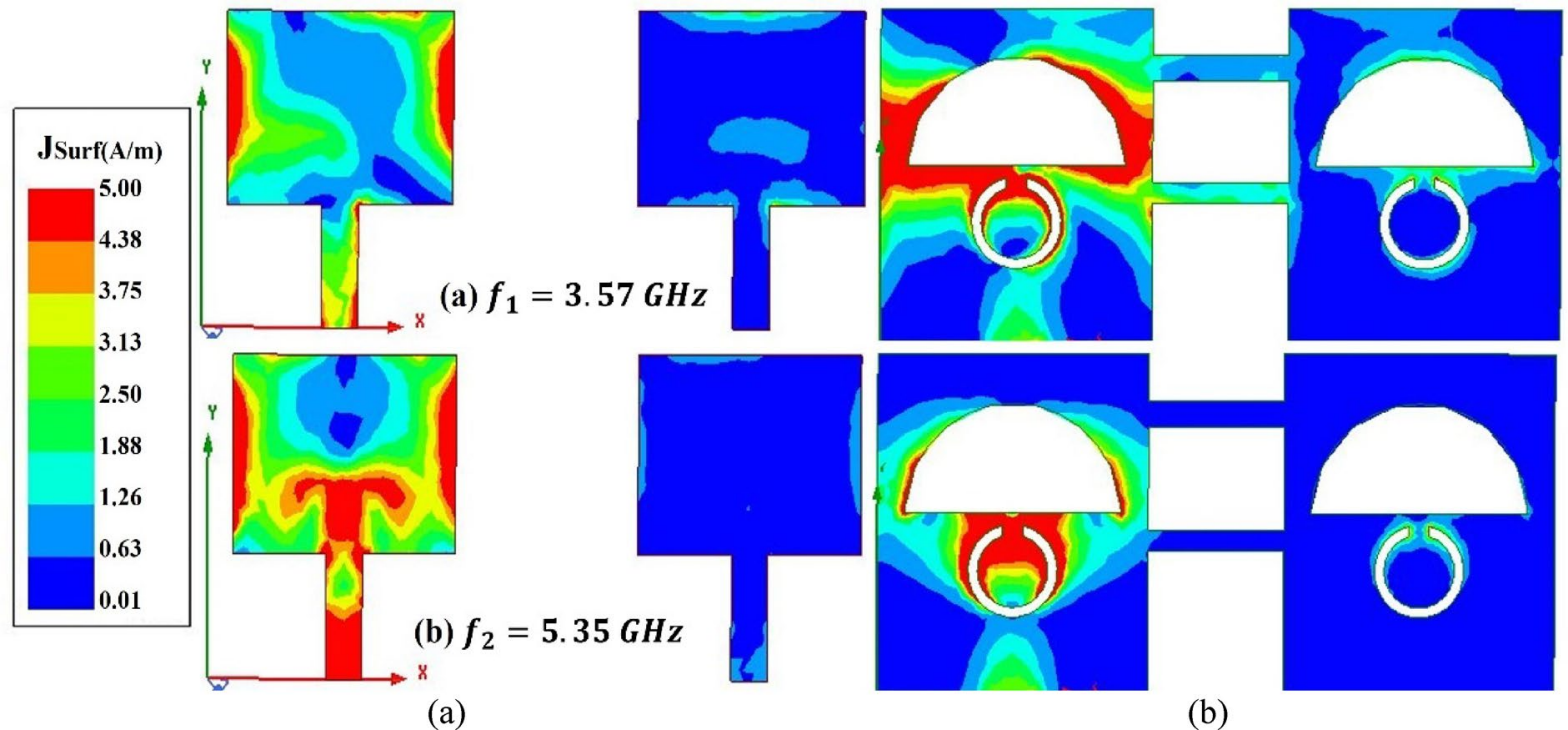
Surface current distribution at 3.57 GHz and 5.35 GHz, showing the patch (right) and ground (left) surfaces, which illustrate the excited resonant modes and isolation mechanism.

When a large amount of the surface current travels from antenna 1 to another antenna, mutual coupling is created among the antennas. To mitigate this effect, it is essential to keep the surface current at the non-energised antenna as low as possible. The interference between the antenna elements can be analysed by examining the surface current depicted in Fig. 11. The current distribution reveals that when only *Port 1* is excited, the maximum current concentration occurs near the edges of the patch at *Port 1*, with minimal coupling to *Port 2*. Similarly, when *Port 2* is excited, the current remains predominantly confined to *Port 2*, indicating effective and reduced coupling between the antennas.

### Equivalent circuit of 2-port MIMO antenna

**Fig. 12:**
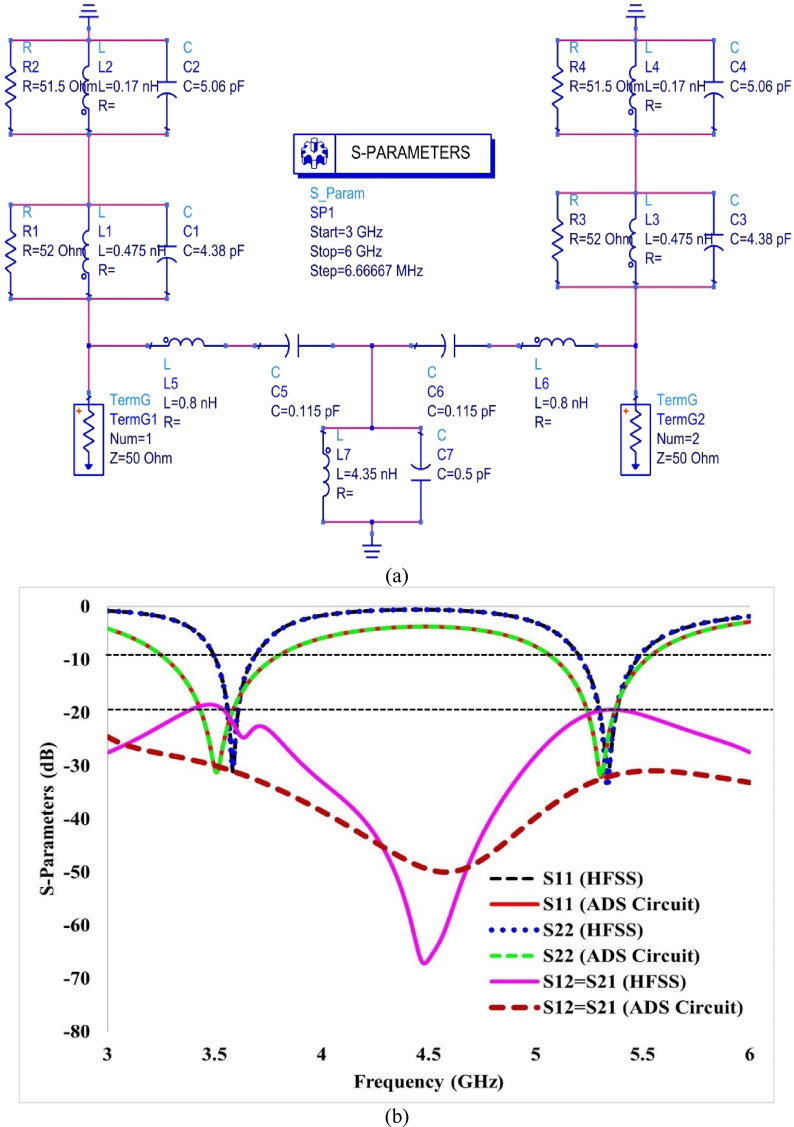
(**a**) Equivalent circuit of the single antenna and (**b**) comparison of S-parameters from HFSS and the equivalent circuit model, showing good agreement and validating the analysis.

The electronic equivalent circuit of the 2-port MIMO antenna is created using the ADS simulator, based on the equivalent circuit of a single antenna, as shown in Fig. 12a. In contrast, Fig. 12b illustrates the comparison of the S-parameters obtained from ADS and HFSS. Two antenna elements are described by the circuit R_1_L_1_C_1_ & R_2_L_2_C_2_ for antenna 1 and R_3_L_3_C_3_ and R_4_L_4_C_4_ for antenna 2. The values of R, L, and C for each antenna element are the same as those of the single antenna element. The coupling between the antenna elements is characterised by two series combinations of C_5_L_5_ and C_6_L_6,_ and ground coupling by a parallel combination of C_7_L_7_.

The optimised values of C_5_ and C_6_ are 0.115 pF, and those of L_5_ and L_6_ are 0.8 nH. The values of L_7_ and C_7_ are 4.35 nH and 0.5 pF, respectively.

### Results and analysis of 2-port MIMO antenna

**Fig. 13:**
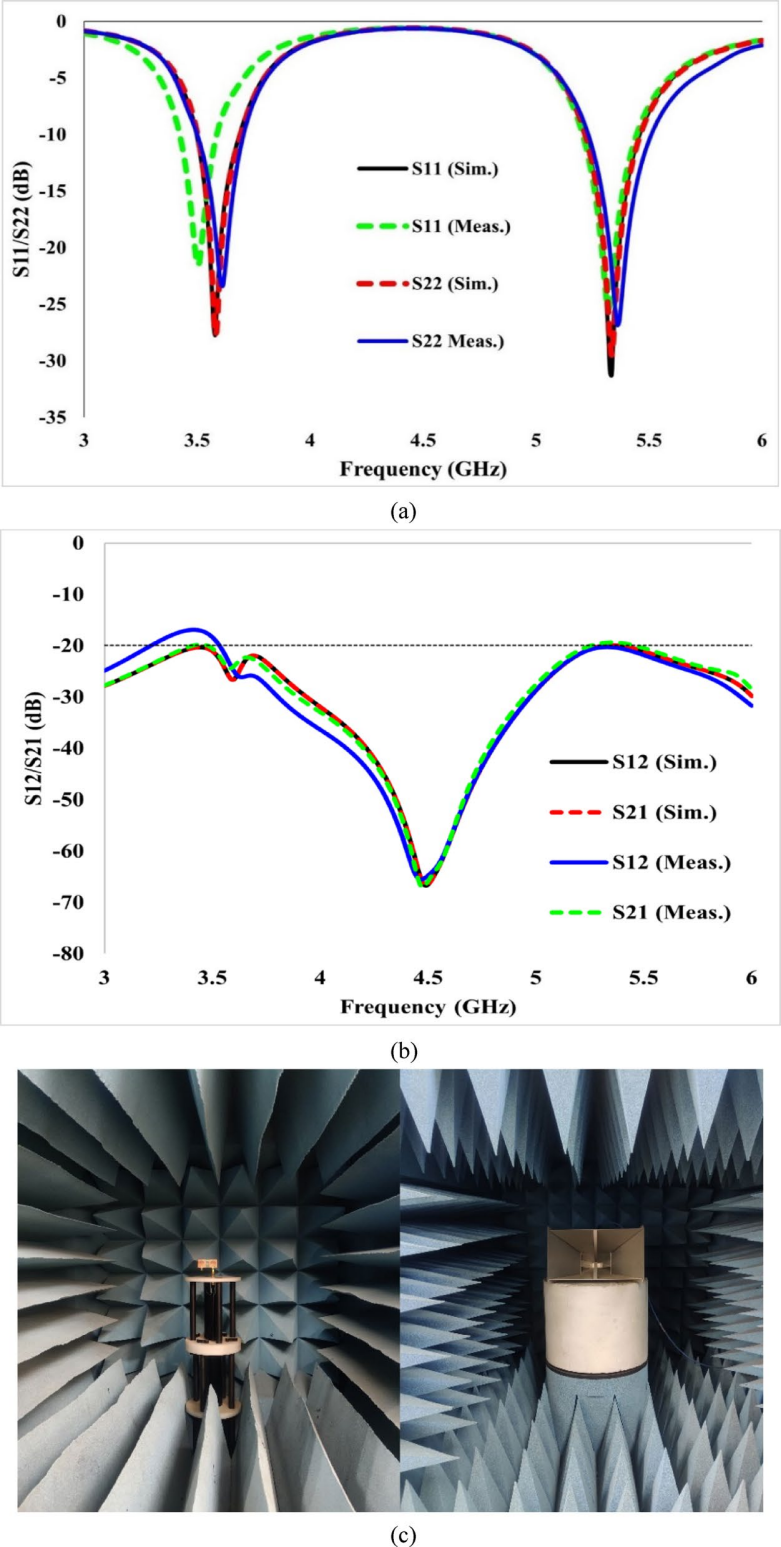
Simulated and tested results of the two-port antenna: (**a**) reflection coefficients, (**b**) transmission coefficients, and (**c**) measurement setup used for validation.

**Fig. 14:**
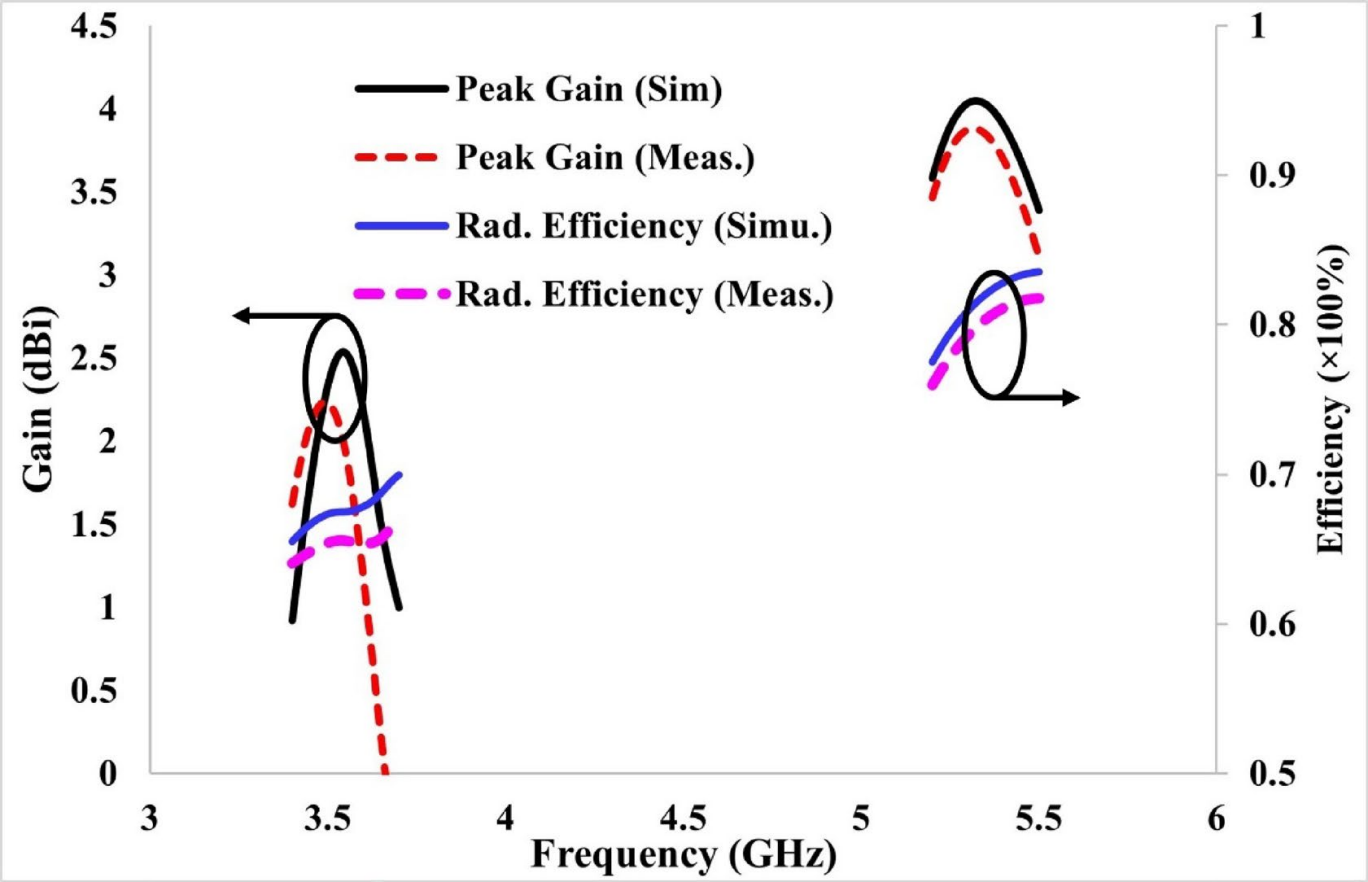
Simulated and tested results of the two-port antenna for gain and efficiency, showing consistent performance between simulation and measurement.

For the validation of the results computed using HFSS, the proposed antenna was constructed and verified using an Agilent Technologies vector network analyzer. The radiation pattern measurement setup is illustrated in Fig. 13c. Figure 13a illustrates the simulated and experimental reflection coefficients of the proposed antenna, which are identical, except for a slight mismatch at the lower port. Similarly, Fig. 13b shows the computed transmission coefficients from HFSS and the measured transmission coefficients of the suggested antenna to observe the inter-element coupling. The antenna-to-antenna coupling is less than − 20 dB, as shown in Fig. 13b. The minor discrepancies between the simulated and measured results are due to fabrication tolerances and variations in the measurement environment. As shown in Fig. 13a, the measured response of antenna-1 exhibits a downward frequency shift, with the resonance observed at 3.50 GHz instead of the expected 3.57 GHz. In addition, the measured S-parameter values are slightly lower compared to the simulated results. A graph for the computed and tested gain and efficiency for the 2-port antenna is shown in Fig. 14. The maximum gain achieved at 3.54 GHz is approximately 2.3 dBi, whereas it reaches a maximum of 4.2 dBi at 5.32 GHz. Radiation efficiency exceeds 65% at 3.54 GHz and 75% at 5.32 GHz, indicating that the developed antenna is highly efficient.

At high frequencies, the dielectric losses may increase due to the use of FR-4. To improve the efficiency and other antenna parameters, substrate materials such as Rogers RO4003, with a low loss tangent of 0.0027, and Rogers RO3003, with a loss tangent of 0.0013, could be used to design the antenna.

**Fig. 15:**
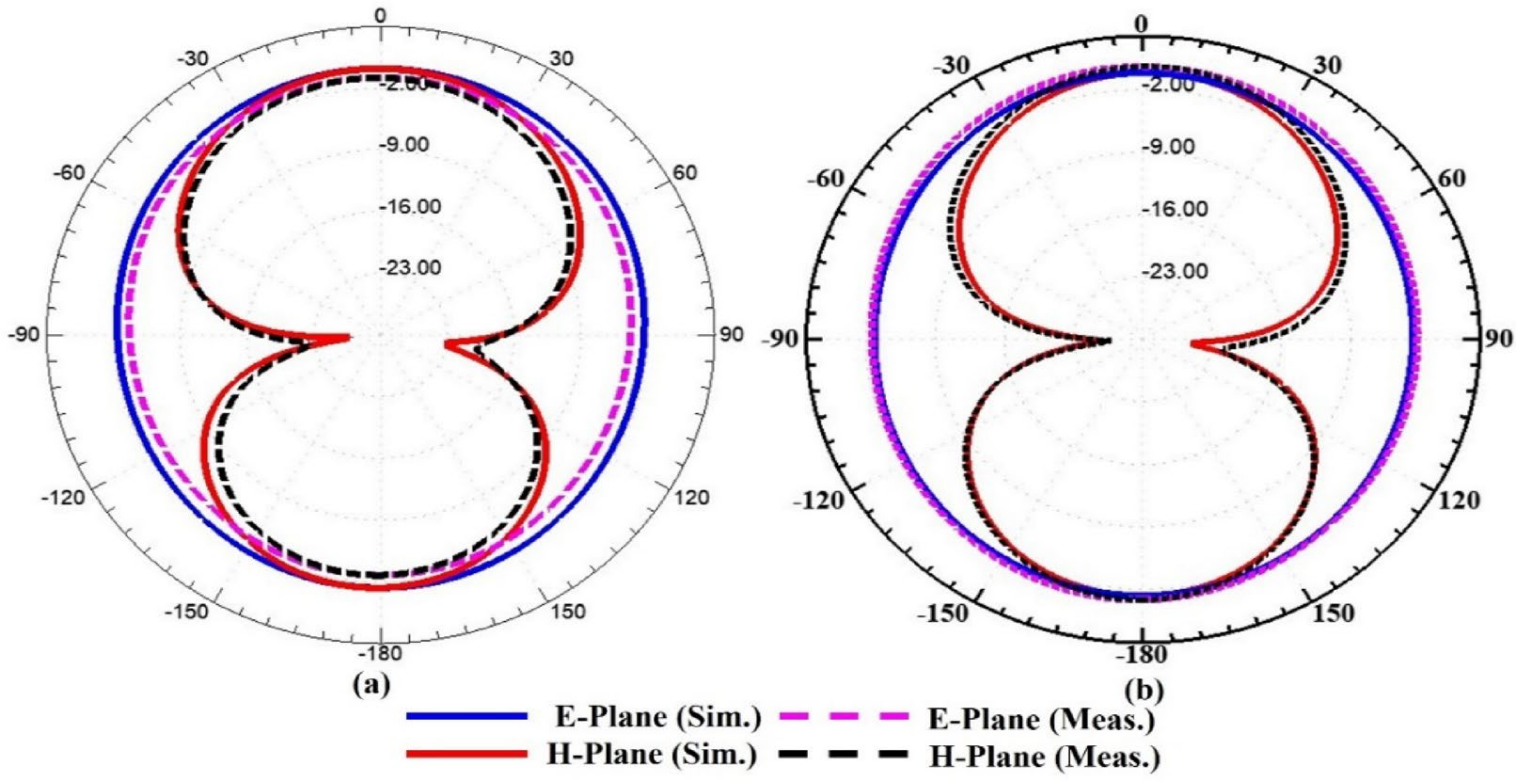
2D simulated and measured radiation patterns of the proposed antenna in the E- and H-planes at (**a**) 3.57 GHz and (**b**) 5.32 GHz, showing close agreement between simulation and measurement.

**Fig. 16:**
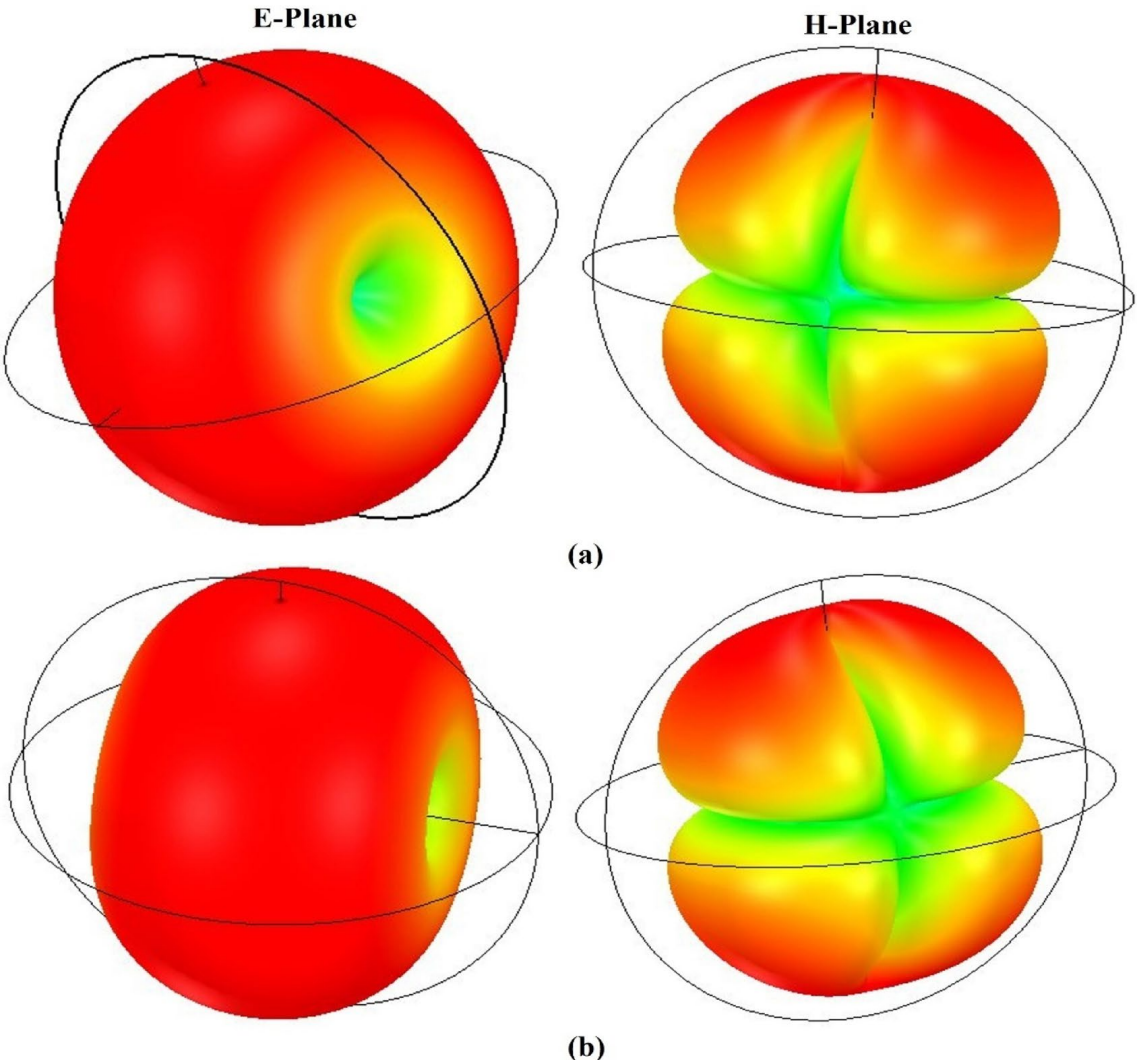
3D simulated radiation patterns of the proposed antenna at (**a**) 3.57 GHz and (**b**) 5.32 GHz, showing an omnidirectional pattern in the E-plane and a bidirectional pattern in the H-plane.

The antenna size can be minimised using a substrate material with a higher εr, but this also results in degrading the performance parameters, such as radiation efficiency. The Q-factor can also be improved with low-loss materials (Rogers RO4003 and 3003), and the antenna is useful for filter and sensing applications. To show the radiation behaviour of the proposed design, the simulated and tested radiation characteristics at 3.50 and 5.32 GHz in both E and H planes are shown in Fig. 15. The antenna exhibits an omnidirectional pattern in the E plane at 3.57 and 5.32 GHz and a bidirectional behaviour in the H plane at both operating frequencies. The radiation patterns at both operating frequencies are acceptable for MIMO antenna systems. Figure 16 illustrates the 3D gain pattern at two frequencies, and it is evident that the antenna exhibits omnidirectional radiation behaviour in the E-plane and bidirectional radiation in the H-plane.

## MIMO antenna diversity parameters

If multiple antenna elements are used, their diversity characteristics must be analysed to ensure optimal performance. This section compares and discusses the diversity characteristics of the MIMO antenna, including the envelope correlation coefficient (ECC), diversity gain (DG), channel capacity loss (CCL), and mean effective gain (MEG), all of which are calculated from simulated and measured S-parameters. Formulas provided in the literature are utilised to determine these parameters.

### ECC and DG

The primary purpose of the MIMO antenna is to provide diversity and enable multiplexing^26^. A significant factor in showing the diversity of the MIMO structure is ECC, as it establishes the degree of similarity between multiple antennas of the MIMO arrangement^27^. The lower the value of ECC, the better the antenna system. The Low value indicates that antennas in the MIMO system are transmitting or receiving signals independently. ECC can be computed using Eq. (9) mentioned in^28^:

9 $${\mathrm{ECC}} = { }\frac{{\left| {{\mathrm{S}}_{11}^{*} {\mathrm{S}}_{12} + {\mathrm{S}}_{21}^{*} {\mathrm{S}}_{22} } \right|}}{{\left( {1 - \left| {{\mathrm{S}}_{11} |^{2} - } \right|{\mathrm{S}}_{21} |^{2} } \right)(1 - \left| {{\mathrm{S}}_{22} |^{2} - } \right|{\mathrm{S}}_{12} |^{2} }}$$

**Fig. 17:**
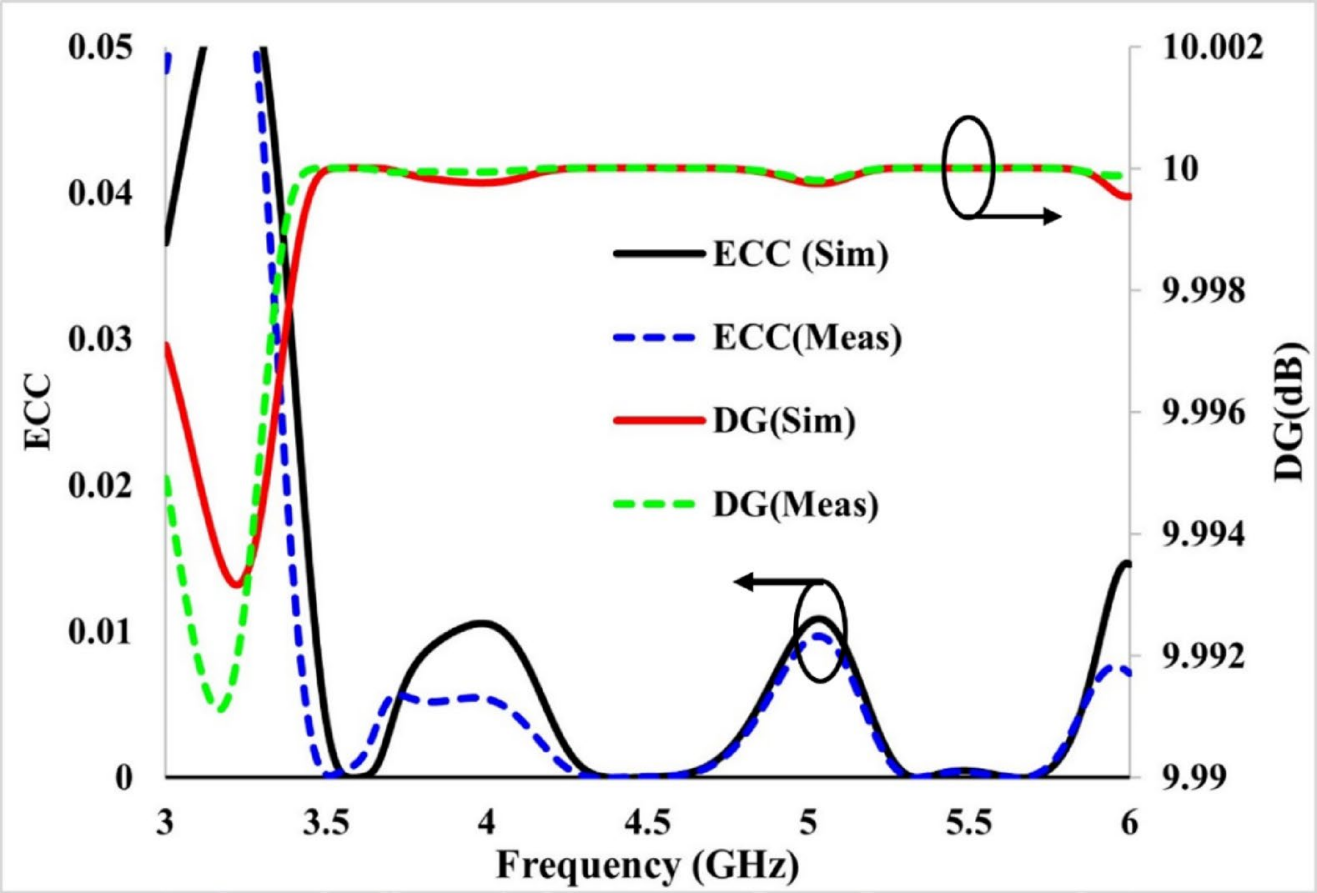
Simulated and experimental envelope correlation coefficient (ECC) of the proposed antenna, showing correlation and diversity performance.

The simulated as well as measured ECC between antenna 1 and antenna 2 is less than 0.002 for both bands, as shown in Fig. 17, which is significantly lower than the specified threshold value of 0.5.

Diversity gain (DG), which indicates the improved signal-to-noise ratio^27^. It means there are various losses in transmission power, and those losses are analysed using DG.

The ideal value of the DG for the MIMO antenna is 10 dB, but due to various environmental conditions, it is often impossible to achieve a DG exactly equal to 10 dB^27,29^. DG is derived using Eq. (10) mentioned in^30^.

10 $$DG = 10 \times \sqrt {1 - (ECC)^{2} }$$

Figure 17 demonstrates that the simulated and tested DG exceeds 9.999 in both operating bands.

### Channel capacity loss (CCL)

Channel capacity loss (CCL) is another critical parameter associated with the MIMO system^31^. Channel capacity is the measure of the maximum amount of information that can be communicated without any loss, and can be measured using Eq. (11) given in^32^.

11 $$CCL = - log_{2} Det\left( {\left| {\beta^{R} } \right|} \right)$$

where

$$\beta^{R} = \left[ {\begin{array}{*{20}c} {\beta_{ii} } & {\beta_{ij} } \\ {\beta_{ji} } & {\beta_{jj} } \\ \end{array} } \right]$$

$\beta_{ii} = 1 - \left[ {\mathop \sum \limits_{j = 1}^{N} |S_{ij} |^{2} } \right]$ and $\beta_{ij} = - \left( {S_{ii}^{*} S_{ij} + S_{ji}^{*} S_{jj} } \right)$ for i, j = 1 or 2.

**Fig. 18:**
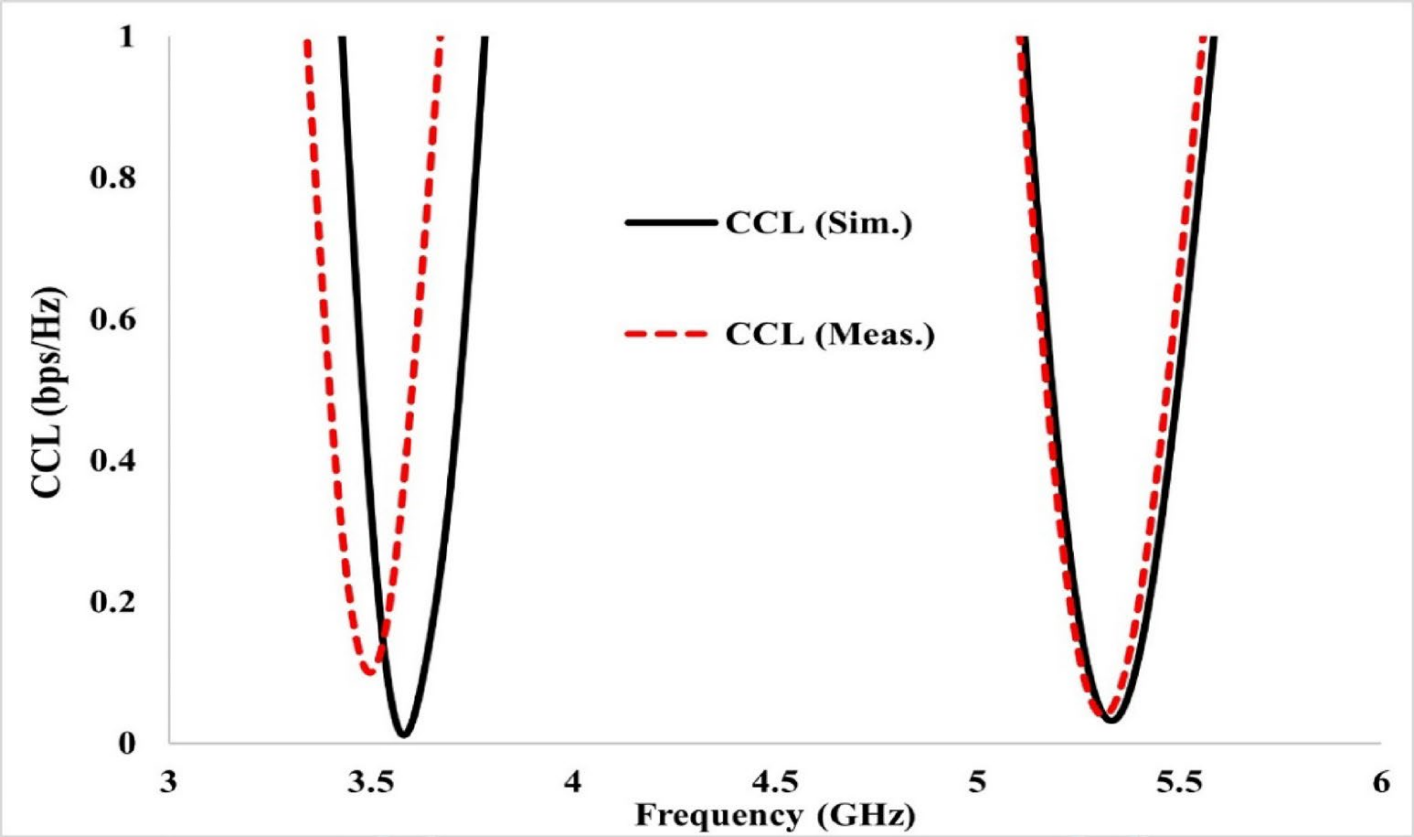
Predicted and experimental channel capacity loss (CCL) analysis showing the maximum achievable information transfer without loss.

For an efficient MIMO antenna, a recommended value of CCL across the entire band is 0.4 bps/Hz^19,33,34^. Figure 18 shows the modelled and tested values of channel capacity loss in bits per second per Hz across both the operating frequency bands. Figure 18 reveals that for the projected antenna, the simulated and experimental value of CCL is found to be below 0.2 bits/Sec/Hz.

### Mean effective gain (MEG) and total active reflection coefficient (TARC)

Mean effective gain (MEG) is used to determine the diversity performance of a MIMO system. MEG shows the amount of power attained by the MIMO antenna system under fading environment conditions^35^ and can be computed by the proportion of the mean received power of the diversity antenna to that of a reference antenna, which is an isotropic antenna, and the range of MEG for an efficient MIMO system is − 12 to − 3 dB. The Eq. (12) given in the reference^36^ is used to calculate the MEG.

12 $$\begin{aligned} MEG_{i} = & \,0.5\left[ {1 - } \right.\left[ {\mathop \sum \limits_{j = 1}^{N} |S_{ij} |^{2} } \right] \\ \left| {MEG_{i} } \right| & \, - \left| {MEG_{j} } \right| \le 3dB \\ \end{aligned}$$

**Fig. 19:**
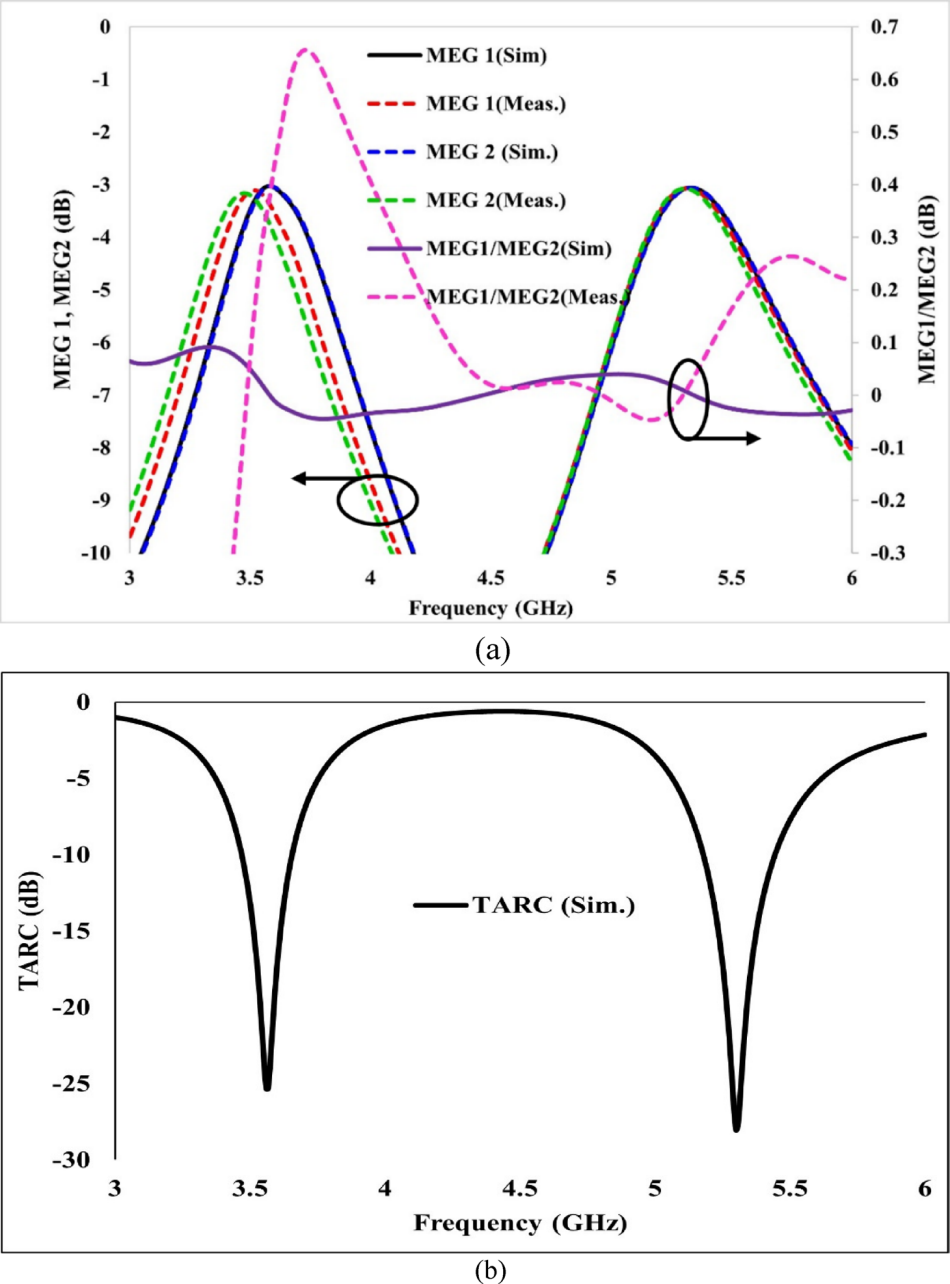
(**a**) Mean effective gain (MEG) analysis of the proposed MIMO antenna, used to determine the diversity performance of the system, (**b**) Simulated TARC.

Figure 19a shows a graph of simulated and measured MEG values, along with their ratio. The predicted and tested MEG value for the presented antenna is nearly − 3 dB, and the difference or ratio of MEG values is less than 1 dB across the two operating bands.

For a MIMO antenna, TARC can be utilised to define the bandwidth and its radiation performance. A TARC value less than − 10 dB indicates minimal power reflections or minimal reflection losses, and it can be considered the reflection coefficient of the entire multiport antenna with all its excited^37,38^. The Eq. (13) mentioned in^39,40^ is used to find the TARC of the proposed Antenna. The TARC for the proposed antenna is plotted in Fig. 19b. From the figure, it is observed that the TARC for the proposed antenna is less than − 10 dB, indicating that the antenna is effective within both operating bands.

13 $$TARC = \sqrt {\frac{{\left| {S_{11} + S_{12} } \right|^{2} + \left| {S_{21} + S_{22} } \right|^{2} }}{2}}$$

At last, Table 5 highlights the comparison of the suggested antenna and existing antennas in the research, focusing on parameters such as size and unit area, number of elements, operating frequencies, isolation, values of ECC, DG, CCL, and maximum gain. The recommended antenna has a smaller size than all the reported two-port MIMO antennas except^41,42^, but their gain is smaller than that of the proposed antenna. The recommended antenna is smaller in size than those referenced in^5,11,13,27,43–47^ Also, the proposed antenna is superior to the antenna reported in^43^ and^11^ in terms of isolation and gain.

**Table 5:** Comparative summary of the suggested antenna against existing MIMO configurations.

Ref. (Year)	Size (mm) (unit area-mm^2^)	No. of ports	Operating frequency range (GHz)	Isolation (dB)	ECC & DG	CCL	Max. gain (dB)	Connected ground (Yes/No)
^5^ (2024)	60 × 120 (3600)	2	3.04–3.09 4.11–4.13 5.18–5.21	> 25	0.014, > 9.99	N.C	3, 4.2	No
^13^ (2024)	66 × 33 (1089)	2	2.1–2.95, 5.1–5.9	22	0.2, N.C	0.21, 0.22	6.2	Yes
^27^ (2025)	60 × 160 (4800)	2	3.58, 5.86, 7.36	> 24	> 9.99	0.001	> 6.7	Yes
^37^ (2025)	60 × 59.8 (1794)	2	2.66–3.82, 4.57–4.91, 6.06–6.50, 7.53–8.08	19.63, 18.6 24.92 13.15	0.02, ~ 10	< 0.4	1.79, 2.66, 4.19, and 3.97	No
^41^ (2024)	36 × 19 (342)	2	3.6 & 7.1	< 20	0.008, > 9.98	< 0.4	2.94, 3.85	Yes
^42^ (2025)	45 × 22 (495)	2	4.36–4.70, 5.60–5.96	> 20	0.0001	0.1	3.7	Yes
^43^ (2023)	56.4 × 36.6 (1032.12)	2	3.37–3.6/ 4.76–5.15/ 6.22–7.27	> 13	< 0.2, N.C	N.C	1.5–6.5	Yes
^44^ (2024)	95 × 52 (2470)	2	2.4, 5.2, 6.0	> 20	0.005,> 9.999	N.C	2.26, 2.53, 1.99	No
^11^ (2022)	62 × 25.6 (793.6)	2	2.99–3.61/ 4.53–4.92	> 16	< 0.002	< 0.32	2.96–3.14/ 3.69–3.84	Yes
^45^ (2025)	23.3 × 66.06 (769.59)	2	3.3–3.78 4.3–5.15	< 23	0.002	0.26	4.9	No
^46^ (2025)	60 × 120 (3600)	2	1.75–2.4	41.9	0.008, 9.99	< 0.4	4.1	Yes
^47^ (2025)	38 × 25 (475)	2	5–6	< 21.5	0.004	0.4	3.5	yes
^48^	60 × 60 (900)	4	3.2–3.8	15	0.16	0.3	4.5	No
^2^ (2024)	73.2 × 73.3 (1341.39)	4	5.85–5.9	> 20		< 0.4	N.C	Yes
^49^ (2025)	39.5 × 39.5 (390.06)	4	4.15–5.25	> 15	< 0.00005, 10	-	6	No
^50^ (2025)	58 × 58 (841)	4	3.15–3.81, 4.31–4.75	17.6	-	-	~3.8	No
^51^ (2024)	48 × 48 (576)	4	4.45–5.2, 5.95–7.15	> 15	< 0.00001	0.03	6.5	Yes
^4^ (2024)	60 × 60 (900)	4	2.20–2.47 3.42–3.54	< 15	< 0.15	< 0.4	3.43, 2.09	Yes
^12^ (2024)	65 × 65 (1056.25)	4	3.1–3.6/5.9–7.1	> 22	0.006	N.C	3.9/4.8	No
Prop. Work	50 × 25 (625)	2	3.50–3.68 5.20–5.46	> 20	< 0.002, ~ 10	< 0.2	2.3, 4.2	Yes

*N.C: not calculated.

The antennas in the references^2,4,12,48–51^ are four-port antennas, but their inter-element isolation is poor, with values less than or equal to 20 dB except for^2^, but it operates over a narrow band from 5.85 to 5.9 GHz with an isolation of more than 20 dB. Similarly, the proposed antenna is superior to the antenna in^48^, in terms of isolation and area of unit element. It is standard practice among researchers working on MIMO antennas to keep the ground planes unconnected, which can produce high isolation between antenna elements because there is no direct current path between the grounds. So, this is not practical to keep the ground planes unconnected for real-time systems^25^. So, the ground planes of the suggested antenna are connected to provide a common reference voltage, while some of the antennas are designed without connecting ground planes.

## Conclusion

The research work presented in this paper aimed to design and fabricate a dual-band two-port antenna that supports 5G applications within the sub-6 GHz frequency range. The antenna geometry began with the design of a single rectangular patch and a modified ground plane. The single antenna element is then escalated to a two-port MIMO antenna. Both the ground planes of the individual antenna structures are connected to boost isolation and minimize interference. The antenna achieves a high Isolation, surpassing − 20 dB in both frequency bands. Also, the antenna provides a gain of 2.3 and 4.2 dBi across both the operating bands. The antenna exhibits a uniform radiation (omnidirectional) characteristic in the E-plane, while displaying a bidirectional pattern in the H-plane. MIMO diversity parameters, such as ECC, DG, CCL, and MEG, have been evaluated to verify the relevance of the antenna for 5G applications. All the parameters across the operating frequencies are within the standard range. The developed antenna is compared with the antennas reported in the previous research works and found to be better in terms of size, gain, and other diversity parameters. All the simulated and experimental results are observed in well-matched conditions. These features confirm the antenna’s suitability for 5G, Wi-Fi, and other wireless applications.

## Supplementary Information

Below is the link to the electronic supplementary material.

Supplementary Material 1

Supplementary Material 2

## Data Availability

The key measured S-parameter Touchstone file supporting the results of this study is included as supplementary material.
